# Lysosomal Dysfunction Is Associated With Intervertebral Disc Degeneration: Multiomics and Machine Learning Identify Molecular Subtypes and Hub Genes

**DOI:** 10.1002/jgm.70109

**Published:** 2026-09-03

**Authors:** Yang Yang, Hong Li, Yixuan Ou, Yaping Yu, Kaiqiang Sun, Shixing Chen, Chenglong Ji

**Affiliations:** ^1^ Department of Orthopedic Surgery Changzheng Hospital, Navy Medical University Shanghai China

**Keywords:** cellular senescence, intervertebral disc degeneration, lithocholic acid, lysosome, machine learning, molecular subtypes

## Abstract

Intervertebral disc degeneration (IVDD) is closely associated with cellular senescence and defective autophagic degradation, but the molecular heterogeneity and functional significance of lysosome‐related alterations remain incompletely understood. We integrated bulk transcriptomic datasets (GSE56081 and GSE70362) and single‐cell RNA‐sequencing data (GSE153066) using consensus clustering, weighted gene coexpression network analysis, machine‐learning algorithms, immune‐signature analysis, and covariate‐adjusted correlation testing. Candidate genes were evaluated in human and rat disc tissues, and PLD3 was further examined by knockdown and overexpression in H_2_O_2_‐treated nucleus pulposus cells (NPCs). Lysosomal function was assessed using LysoTracker staining, cathepsin activity, and autophagy‐related markers, and the preclinical effects of lithocholic acid (LA) were evaluated in cultured NPCs and a rat needle‐puncture model. Two lysosome‐related molecular subtypes were identified: a senescence/inflammation‐enriched subtype and a metabolism‐enriched subtype. The lysosomal gene‐signature score was positively associated with the senescence score after adjustment for total cellular transcript counts (partial *r* = 0.422; empirical permutation *p* = 0.0005), supporting coordinated transcriptional activation rather than enhanced degradative function. HYAL1, MMD, PLD3, and ANK3 were prioritized as candidate hub genes. PLD3 knockdown aggravated H_2_O_2_‐induced lysosomal impairment, matrix degeneration, and senescence, whereas PLD3 overexpression produced opposing protective effects. LA partially improved acidic lysosomal compartments, cathepsin activity, autophagic degradation, and senescence‐associated changes in vitro and attenuated degeneration‐associated histological and molecular alterations in vivo. These findings reveal distinct lysosome‐related phenotypes in IVDD, functionally support PLD3 as a contributor to lysosomal homeostasis, and suggest that lysosome‐modulating interventions may have therapeutic potential.

## Graphical abstract

Intervertebral disc degeneration (IVDD) is linked to lysosomal dysfunction, impaired autophagic degradation, and cellular senescence. Integrating bulk and single‐cell transcriptomics with machine learning, this study identified two lysosome‐related molecular subtypes and four hub genes: HYAL1, MMD, PLD3, and ANK3. PLD3 supported lysosomal homeostasis, as its loss aggravated oxidative stress‐induced degeneration and senescence, whereas overexpression was protective. Lithocholic acid partially restored lysosomal function and reduced degenerative changes in cultured nucleus pulposus cells and a rat IVDD model, supporting lysosome‐targeted therapy as a potential strategy.

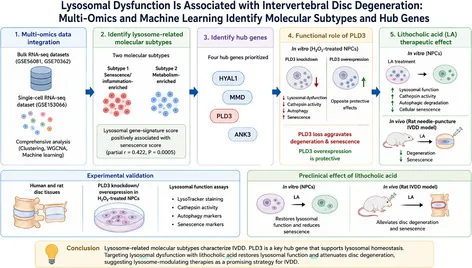

**Abbreviations**
ARRIVE: Animal Research: Reporting of In Vivo Experiments
AUC: area under the curve
DCA: decision curve analysis
GO: Gene Ontology
GSEA: gene set enrichment analysis
H&E: hematoxylin and eosin
IACUC: Institutional Animal Care and Use Committee
IVDD: intervertebral disc degeneration
KEGG: Kyoto Encyclopedia of Genes and Genomes
LA: lithocholic acid
LASSO: least absolute shrinkage and selection operator
LRGs: lysosome‐related genes
NPCs: nucleus pulposus cells
PBS: phosphate‐buffered saline
ROC: receiver operating characteristic
SD: Sprague–Dawley
SF: safranin O/fast green
SHAP: SHapley Additive exPlanations
ssGSEA: single‐sample gene set enrichment analysis
UMAP: Uniform Manifold Approximation and Projection
WGCNA: weighted gene coexpression network analysis

## 1 Introduction

Intervertebral disc degeneration (IVDD) is a leading cause of chronic low back pain and physical disability worldwide, imposing a substantial socioeconomic and healthcare burden [1]. Low back pain affects over 600 million people globally and is the primary contributor to years lived with disability, with IVDD serving as one of its most common structural causes [2, 3]. Epidemiologically, the prevalence of IVDD increases markedly with age, affecting more than 40% of individuals by the age of 40 and over 80% by age 60 [2]. Despite its high prevalence, current treatments remain largely palliative, focusing on symptom management rather than targeting underlying molecular pathology [4]. Although ageing, mechanical overloading, and inflammatory cascades are widely recognized as key contributors, the precise cellular and molecular drivers of IVDD progression are still incompletely understood.

Lysosomes serve as central hubs within cells for the degradation and recycling of damaged or obsolete macromolecules and organelles. In response to stressors including nutrient deprivation, lysosomes play a pivotal role in regulating endocytosis, phagocytosis, and the self‐degradative process of autophagy [5]. Previous studies have underscored the crucial role of lysosomal function in maintaining cellular homeostasis. Lysosomes are pivotal for regulating cellular quiescence and are asymmetrically partitioned during hematopoietic stem cell division. A study by Tasleem Arif et al. revealed that lysosomes were hyperacidic, damaged, and dysfunctional in old hematopoietic stem cells [6]. Lysosomal impairment has also been implicated in neurodegenerative disorders such as Alzheimer's disease, where defective lysosomal acidification contributes to protein aggregation and neuronal loss [7]. Within the musculoskeletal field, studies have highlighted that lysosomal function declines with age in chondrocytes, contributing to osteoarthritis progression through impaired autophagy and enhanced senescence [8]. Notably, modulating lysosomal activity, for example, via TFEB activation or v‐ATPase regulation, has shown promise in rejuvenating aged stem cells and delaying tissue degeneration, positioning the lysosome as a compelling therapeutic target in age‐related disorders [9]. Given the critical role of senescence and autophagy in mediating IVDD, we hypothesized that impaired lysosomal activity disrupts autophagic degradation, triggering senescence‐associated secretory phenotypes (SASP) and inflammatory responses during IVDD. Although in the intervertebral disc (IVD), emerging studies suggest that lysosomal dysfunction may underlie the degenerative cascade, a systematic molecular classification of IVDD based on lysosomal pathways, along with the identification of key regulatory genes and their therapeutic relevance, remains largely unexplored.

In the present study, we integrated bulk transcriptomics and single‐cell RNA sequencing (scRNA‐seq) with machine learning approaches to delineate the landscape of lysosomal dysfunction in IVDD. We first identified lysosome‐related molecular subtypes, uncovered hub lysosome‐related genes (LRGs) with diagnostic and functional significance, and characterized their immune and senescence‐associated networks. We further explored the effects of LA as a lysosome‐modulating intervention in cellular and animal models. Our findings provide new insights into the lysosome–senescence axis in IVDD, identify potential biomarkers, and provide preliminary preclinical support for lysosomal homeostasis as a possible therapeutic target.

## 2 Methods and Materials

### 2.1 scRNA‐seq Data Analysis

scRNA‐seq data were processed and analyzed using the Seurat package [10]. Low‐quality cells were removed by filtering out outliers in gene counts, as these typically represent apoptotic cells, doublets, or cells with compromised membrane integrity. After quality filtering, high‐quality cells were normalized and scaled through the “NormalizeData” and “ScaleData” functions to apply linear transformation. Dimension reduction and visualization were carried out using Uniform Manifold Approximation and Projection (UMAP). Cell clusters were identified based on known marker gene expression patterns.

### 2.2 Calculation of Senescence Gene‐Signature Scores

Senescence‐related and lysosome‐related transcriptional enrichment at the single‐cell level was quantified using the AUCell algorithm [11, 12]. Senescence hallmark gene sets were retrieved from the MSigDB database (https://www.gsea‐msigdb.org). Based on these gene sets, the senescence gene‐signature score and corresponding AUC (area under the curve) value were calculated for each NPC subtype.

### 2.3 Lysosomal Gene‐Signature Scoring

Lysosome‐related transcriptional enrichment at the single‐cell level was quantified using the AUCell algorithm and a curated LRG set. The resulting lysosomal gene‐signature score reflects the relative expression and enrichment of lysosome‐associated genes and may indicate transcriptional activation or lysosomal biogenesis. Importantly, this score does not directly measure lysosomal acidification, hydrolase activity, autophagic substrate degradation, or overall degradative capacity. Therefore, it was interpreted as a transcriptional signature rather than a direct functional measurement.

### 2.4 Partial Correlation and Permutation Analysis

To assess whether the association between the lysosomal gene‐signature score and the senescence score was confounded by overall cellular transcriptional activity, partial correlation analysis was performed using nCount_RNA, defined as the total number of unique molecular identifier counts per cell, as a covariate. The partial correlation coefficient between the lysosomal gene‐signature score and the senescence score was calculated after adjustment for nCount_RNA.

To further evaluate the specificity of the adjusted association, a random permutation test was performed with 2000 iterations. In each iteration, the correspondence between the lysosomal gene‐signature score and the senescence score was randomly permuted, and the partial correlation coefficient was recalculated while controlling for nCount_RNA. The resulting coefficients were used to construct an empirical null distribution. The permutation *p* value was calculated as the proportion of permuted partial correlation coefficients that were equal to or greater than the observed coefficient.

Partial correlation analysis was used to evaluate the association between the lysosomal and senescence gene‐signature scores after adjustment for total UMI counts per cell (nCount_RNA). The robustness of the adjusted association was further assessed using a 2000‐iteration random permutation test. Empirical permutation *p* values were calculated using the (*k* + 1)(*N* + 1) correction to avoid reporting a *p* value of zero.

### 2.5 Data Acquisition and Processing

In this study, transcriptomic data were acquired from the Gene Expression Omnibus (GEO) database (https://www.ncbi.nlm.nih.gov/geo/). Publicly available microarray expression datasets (accession numbers: GSE56081 and GSE70362) and single‐cell RNA‐sequencing (scRNA‐seq) data (accession number: GSE153066) were downloaded and processed for subsequent analyses [13, 14]. To integrate the two bulk RNA‐sequencing datasets (GSE56081 and GSE70362), we performed batch effect correction using the “ComBat” algorithm from the “sva” R package based on previous study [15]. ComBat is an empirical Bayes‐based method widely recognized for its robustness in adjusting for batch effects in gene expression data while preserving biological variation of interest. Prior to correction, data were normalized using the “voom” function from the “limma” package. The batch variable was defined as the dataset origin (GSE56081 vs. GSE70362), and the correction was applied with parametric adjustment mode. To estimate the batch effect in the merged data, principal component analysis (PCA) was used to visualize the data. A total of 879 LRGs were curated based on established references (Table S1).

### 2.6 Differential Expression Gene Analysis and Functional Analysis

Differentially expressed genes (DEGs) in the GSE56081 and GSE70362 datasets were identified using the “limma” R package [16], with a significance threshold set at *p* < 0.05. Result visualization was performed using the “pheatmap” package to generate volcano plots and heatmaps. Functional annotation of DEGs was carried out with the “org.Hs.eg.db” and “clusterProfiler” R packages [17]. Gene Ontology (GO) and Kyoto Encyclopedia of Genes and Genomes (KEGG) enrichment analyses were conducted to identify significantly enriched biological terms, using a threshold of *p* < 0.05. GO analysis covered three categories: biological process (BP), molecular function (MF), and cellular component (CC).

### 2.7 Weighted Gene Coexpression Network Analysis (WGCNA)

WGCNA was performed using the WGCNA R package to construct gene coexpression networks associated with clinical traits [18]. To enhance result accuracy, the top 25% of genes exhibiting the highest variance from the combined dataset were initially selected as input. The optimal soft‐thresholding power was determined using the scale‐free topology criterion. Subsequently, a weighted adjacency matrix was constructed and transformed into a topological overlap matrix (TOM). Gene modules were identified via hierarchical clustering, with those containing more than 50 genes being retained for further analysis and each assigned a distinct color for visualization. Module eigengenes (MEs), representing the first principal components of individual modules, were computed to summarize module expression profiles. Module significance (MS) was quantified as the absolute correlation coefficient between each ME and the relevant clinical traits. Concurrently, gene significance (GS) was defined for each gene as its correlation with the clinical phenotype of interest.

### 2.8 SHAP Method

For hub gene selection, we employed three widely adopted machine learning algorithms, CatBoost, XGBoost, and LightGBM, as predictive models. Their application in biomedical research is supported by notable advantages in flexibility, scalability, and ease of implementation [19, 20, 21]. The hyperparameters of each predictive model were set as follows: for CatBoost, learning rate = 0.1, depth = 4, l2_leaf_reg = 5, subsample = 0.6, early stopping_rounds = 20, and bootstrap_type = Bernoulli; for LightGBM, Dist = k_categorical, Score = LogScore, Base = default_tree_learner, n_estimators = 1000, learning_rate = 0.01, minibatch_frac = 1.0, col_sample = 1.0, and tol = 0.0001; for XGBoost, eta = 0.3, max_depth = 6, gamma = 0, lambda = 1, alpha = 0, subsample = 1, and colsample_bytree = 1. To interpret the contribution of individual features to the model predictions, we further utilized SHAP (SHapley Additive exPlanations), a game theory–based interpretability framework [22]. After model training, SHAP analysis was performed to identify the genes exerting the greatest influence on predictive outcomes.

### 2.9 Immune Infiltration Analysis

The infiltration levels of 28 immune cell subtypes were assessed via single‐sample gene set enrichment analysis (ssGSEA), as previously described [23]. Differences in the ssGSEA enrichment scores of 28 immune‐cell signatures between groups were assessed using the Wilcoxon rank‐sum test. To account for multiple comparisons, raw *p* values from all 28 comparisons were adjusted using the Benjamini–Hochberg false discovery rate method. Bonferroni‐adjusted *p* values were additionally calculated as a conservative sensitivity analysis. An FDR‐adjusted *q* value < 0.05 was considered statistically significant. Because ssGSEA scores represent relative enrichment of transcriptional signatures rather than absolute immune‐cell proportions, the immune infiltration results were interpreted as exploratory.

### 2.10 Construction and Validation of Nomogram Models

Hub genes were used to construct nomograms via the “rms” R package. The predictive accuracy of the nomogram was evaluated using calibration curves, and its clinical utility was assessed through decision curve analysis (DCA). To evaluate the diagnostic performance of the identified LRGs, receiver operating characteristic (ROC) curves were generated for each gene individually using the “pROC” R package [24].

### 2.11 Histological Analysis of Human or Rat IVD Tissue

Human nucleus pulposus (NP) tissues were obtained from patients undergoing spinal surgery for spinal fracture, tethered cord syndrome, or lumbar disc herniation at our institution. The degree of IVDD was assessed based on the Pfirrmann classification system through evaluation of T2‐weighted magnetic resonance imaging (MRI) scans [25]. Due to the limited availability of truly healthy disc samples, tissues classified as Pfirrmann grade II were defined as the relatively normal control group, whereas those with grades III to V were assigned to the IVDD group.

For histological assessment, human NP tissues were meticulously dissected from fresh surgical samples to exclude annulus fibrosus (AF) and other non‐NP tissue, and then fixed with 4% paraformaldehyde (Servicebio, G1101) before paraffin embedding and sectioning. By contrast, whole IVDs were collected from rats, followed by fixation in 4% paraformaldehyde, paraffin embedding, and subsequent staining procedures. Sections (4 μm in thickness) were stained using hematoxylin and eosin (H&E) or safranin O/fast green (SF) according to established protocols, and images were acquired under a light microscope (Olympus, Japan).

All MRI‐based Pfirrmann grading and histological evaluations were performed independently by two investigators blinded to group allocation. Discrepancies were resolved through consensus discussion.

### 2.12 Quantitative Reverse Transcription Polymerase Chain Reaction Analysis

qRT‐PCR was performed according to standardized protocols. Total RNA was extracted from human or rat IVD tissues using the Rapture Universal RNA Plus Kit (R4013‐02; Magen, China), following the manufacturer's instructions. Purified RNA was reverse‐transcribed into cDNA, which was subsequently amplified using ChamQ Universal SYBR qPCR Master Mix (Q711‐03; Vazyme, China). GAPDH served as the internal reference gene, and relative gene expression levels were calculated using the 2^−ΔΔCt^ method.

### 2.13 Isolation and Culture of Primary Rat NPCs

The primary isolation and culture of NPCs were performed following a previously established protocol. Briefly, male SD rats (200 g) were obtained from our institutional animal center [11, 25]. Following euthanasia, the tail was excised, immersed in 70% ethanol for 15 min for disinfection, and aseptically transferred into a laminar flow hood. The skin was carefully dissected away using sterile scissors to expose the IVDs. Under microscopic guidance, the distinct white annular structure of the disc was identified, after which the AF was incised with a sterile surgical blade to release the pressurized NP tissue. The freshly extruded gelatinous NP material was collected and subjected to sequential enzymatic digestion. Initially, it was incubated with 0.25% trypsin–EDTA (Servicebio, G4021) for 30 min, followed by digestion in 0.2% collagenase II (Servicebio, GC305014) for an additional 30 min in a gently agitated 37°C water bath. After digestion, the isolated nucleus pulposus cells (NPCs) were washed and resuspended in DF‐12 culture medium (Servicebio, G4612) containing 10% fetal bovine serum (ZETAlife, cat. no. Z7010FBS) and 1% penicillin–streptomycin (Gibco, cat. no. 15140163). Cells at passage 0 were cultured in a humidified incubator at 37°C with 5% CO_2_. Upon reaching approximately 90% confluence, cells were harvested for subsequent experimental use.

### 2.14 The Establishment of Rat NPC Senescence Model Induced by H_2_O_2_

Rat NPCs were seeded in 12‐well plates at a density of 5 × 10^4^ cells per well and allowed to adhere for 24 h. To induce cellular senescence, cells were exposed to 100‐μM H_2_O_2_ in complete culture medium for 24 h under standard culture conditions. A parallel control group received PBS treatment under identical conditions. Following treatment, the induction of senescence was evaluated by IF staining for the senescence‐associated markers p16ᴵᴺᴷ^4^ᵃ.

### 2.15 LysoTracker Staining

Lysosomal acidic compartments were assessed using LysoTracker Red (IF1840, Solarbio) according to the manufacturer's instructions. After the indicated treatments, NPCs were incubated with LysoTracker probe at 37°C for 30 min, washed with PBS, and immediately imaged using a fluorescence microscope. Fluorescence intensity was quantified using ImageJ. Because LysoTracker fluorescence can be affected by both lysosomal acidity and lysosome abundance, the results were interpreted together with cathepsin activity and autophagic degradation‐related measurements.

### 2.16 Cathepsin Activity Assay

After the indicated treatments, NPCs were washed twice with ice‐cold PBS, collected, and lysed on ice using the lysis buffer supplied with the Cathepsin B Activity Fluorometric Assay Kit (E‐bc‐F059; Elabscience, Wuhan, China). The cell lysates were centrifuged at 10,000 × *g* for 10 min at 4°C, and the resulting supernatants were collected. An aliquot of each supernatant was used to determine the total protein concentration, whereas the remaining supernatant was kept on ice for the activity assay. Cathepsin B activity was measured strictly according to the manufacturer's instructions. Fluorescence was recorded using a SpectraMax iD5 microplate reader (Molecular Devices, San Jose, CA, USA) at an excitation wavelength of 355 nm and an emission wavelength of 460 nm. The plate was shaken for 5 s and incubated at 37°C in the dark for 10 min between fluorescence measurements. The change in fluorescence intensity was used to calculate cathepsin B activity, which was normalized to the total protein concentration and expressed relative to the control group. Three independent biological replicates were analyzed for each group, with three technical replicates per sample.

### 2.17 PLD3 Knockdown and Overexpression in NPCs

Primary rat NPCs were transfected with PLD3‐specific siRNA or a PLD3 overexpression plasmid (BioTNT, Shanghai) using Lip3000 (L3000015, Invitrogen) according to the manufacturer's instructions. Corresponding non‐targeting siRNA and empty‐vector controls were used to control for nonspecific effects of transfection. PLD3 knockdown and overexpression efficiencies were confirmed using qRT‐PCR. At 24 h after transfection, the cells were exposed to 100‐μM H_2_O_2_ for 24 h. The experimental groups included the untreated control group, H_2_O_2_ group, H_2_O_2_ plus PLD3 knockdown group, and H_2_O_2_ plus PLD3 overexpression group. Lysosomal acidic compartments were evaluated using LysoTracker staining, and lysosomal proteolytic capacity was assessed using a fluorometric Cathepsin B Activity Assay Kit (E‐bc‐F059, Elabscience). The expression levels of the matrix anabolic marker *Acan*, catabolic marker *Mmp13*, and senescence‐associated markers *Cdkn1a* were determined by qRT‐PCR.

### 2.18 Establishment of the Rat IVDD Model and Local LA Administration

All animal experiments were conducted in compliance with the ARRIVE guidelines. An established needle puncture‐induced model of IVDD was generated using 8‐week‐old male Sprague–Dawley (SD) rats following a previously described protocol [26]. Briefly, rats were anesthetized with isoflurane delivered through a precision vaporizer, using an induction chamber followed by a nose cone for maintenance. Anesthesia was induced with 3%–4% isoflurane in 100% oxygen at a flow rate of 1.0 L/min and maintained with 1.5%–2% isoflurane in 100% oxygen at 0.8 L/min during surgery. After a stable plane of anesthesia was achieved, a 22‐gauge needle was vertically inserted into the coccygeal IVD at Co5/6 and rotated for 15 s to induce disc injury. For the experimental pharmacological intervention, LA was locally administered into the punctured disc at a nominal concentration of 10 μM in an injection volume of 3 μL per disc, corresponding to approximately 30 pmol of LA per disc. The concentration was selected based on a preliminary in vitro concentration–response and cell‐viability assay, in which 10‐μM LA produced the highest relative cell‐viability signal among the tested concentrations. The injection volume was selected based on preliminary procedural optimization and experience from our previous studies [11, 25]. The IVDD control group received an equivalent volume of phosphate‐buffered saline (PBS). Injection volumes exceeding 5 μL were avoided because visible leakage frequently occurred during needle withdrawal, potentially reducing the consistency of local delivery [11, 25]. Therefore, an injection volume of 3 μL was used to facilitate reproducible intradiscal administration while minimizing visible leakage. The stated concentration represents the nominal concentration of the injected solution; the actual intradiscal concentration, spatial distribution, and retention time of LA were not measured in the present study.

At 4 weeks post‐operation, all rats were humanely euthanized by carbon dioxide (CO_2_) inhalation in a dedicated euthanasia chamber, with a gradual fill method with 20%–30% chamber volume displacement per minute to minimize distress, followed by secondary confirmation via cervical dislocation. All procedures were performed under deep anesthesia induced by isoflurane (3%–4% in 100% oxygen) to ensure unconsciousness prior to euthanasia. Disc tissues were then collected for subsequent histological and molecular analyses.

### 2.19 Ethics Approval

All animal experiments were conducted in strict accordance with the guidelines of our Institutional Animal Care and Use Committee (IACUC) and were approved by the Institutional Review Board of our hospital (2025H012‐A03). The use of human NP tissues was approved by the Ethics Committee of our Hospital (2026Q056‐1). All patients provided written informed consent prior to participation.

### 2.20 Statistical Analysis

All quantitative data are presented as mean ± standard deviation (SD). Differences between two groups were assessed using the unpaired Student's *t*‐test for normally distributed data, and the Mann–Whitney *U* test for nonnormally distributed data. Comparisons across multiple groups were performed using one‐way analysis of variance (ANOVA). Statistical analyses were conducted with GraphPad Prism (Version 9.0), and a *p* value < 0.05 was considered statistically significant.

## 3 Results

### 3.1 Consensus Clustering and Differential Expression Analysis Identified Lysosome‐Related Transcriptional Subtypes in IVDD

**FIGURE 1:**
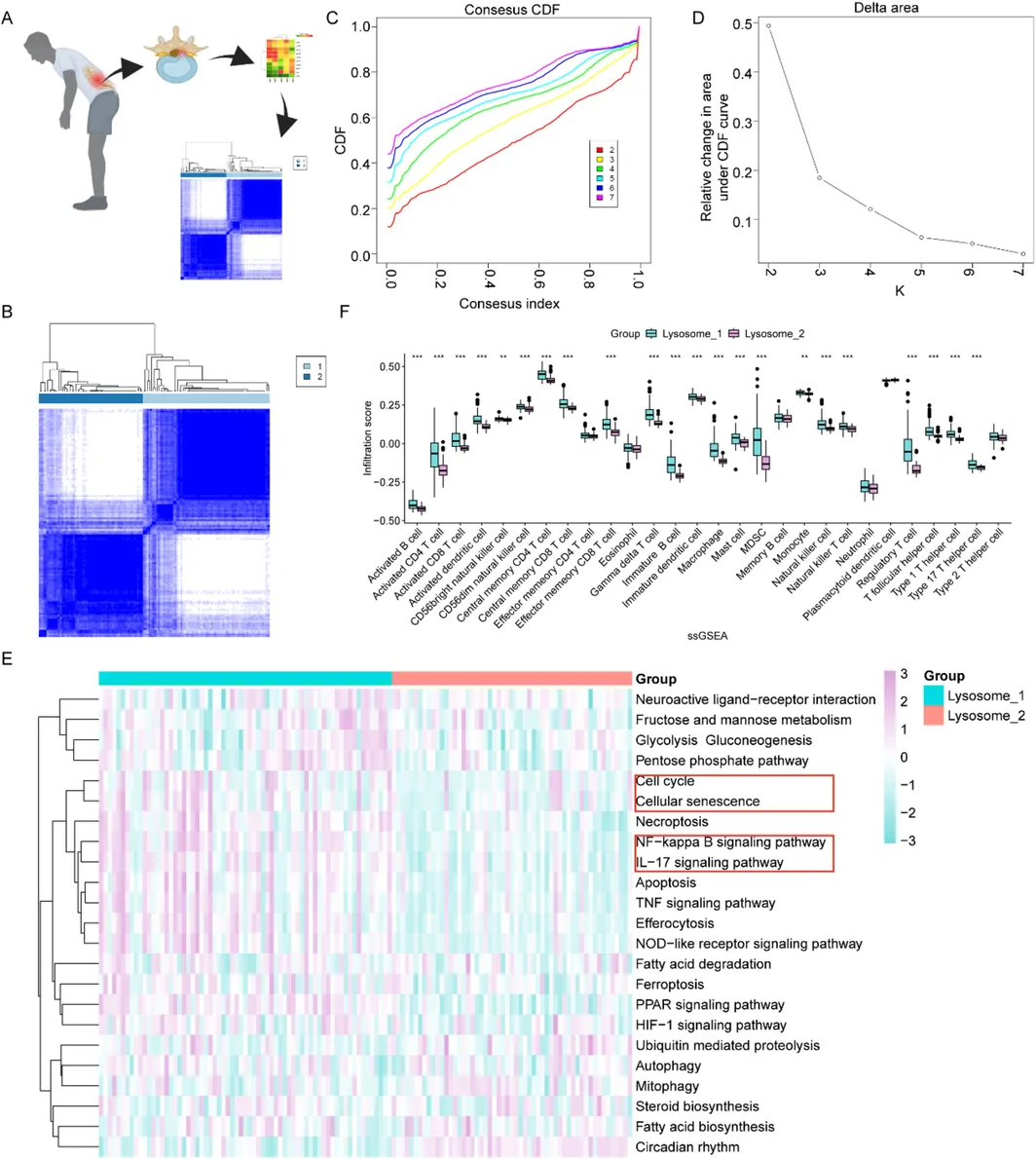
Consensus clustering and differential expression analysis based on LRGs in IVDD patients. (A) Schematic workflow for identifying lysosome‐related molecular subtypes in IVDD. (B) Consensus clustering matrix for IVDD patients at *k* = 2 (*n* = 122). (C) Cumulative distribution function (CDF) curves for *k* = 2–7. (D) Relative change in the area under the CDF curve for *k* = 2–7. (E) Gene set variation analysis (GSVA) of pathways differentially enriched between Cluster 1 and Cluster 2. Colors represent normalized enrichment scores. (F) ssGSEA‐based enrichment scores of 28 immune‐cell signatures in the two clusters. Statistical significance was assessed using the Wilcoxon rank‐sum test; boxes indicate the interquartile range and whiskers indicate 1.5 × the interquartile range (*n* = 61 per cluster). *p < 0.05, **p < 0.01, and ***p < 0.001. Abbreviations: CDF, cumulative distribution function; GSVA, gene set variation analysis; IVDD, intervertebral disc degeneration; ssGSEA, single‐sample gene set enrichment analysis.

To characterize the association between lysosome‐related transcriptional programs and IVDD, we performed consensus clustering of 122 NP tissue samples obtained from 108 patients based on LRG expression to identify distinct lysosome‐associated molecular subtypes (Figure 1A). The data was downloaded from GSA repository (https://ngdc.cncb.ac.cn/gsa‐human, accession code: HRA009301) [27]. Notably, to evaluate lysosomal status at the single‐cell level, we calculated a “lysosomal gene signature score” based on the expression of LRG. It is important to note that this score reflects transcriptional activity and lysosomal biogenesis, which may increase as a compensatory response to stress but does not directly measure lysosomal degradative capacity or functional homeostasis. As the results show, the optimal cluster number was determined to be *k* = 2, as this partition demonstrated the highest stability and reliability (Figure 1B). Differences between adjacent clustering solutions (*k* = 3–7) were further visualized using the cumulative distribution function (CDF) curve area analysis (Figure 1B–D). Gene set variation analysis revealed two biologically distinct lysosome‐related molecular phenotypes. Cluster 1 was enriched in cellular senescence, cell‐cycle dysregulation, and inflammatory signaling pathways (Figure 1E). In contrast, Cluster 2 was characterized by enrichment of steroid biosynthesis, fatty‐acid biosynthesis, and circadian‐rhythm pathways and was designated the metabolism‐enriched subtype (Figure 1E). These findings indicate that IVDD exhibits substantial molecular heterogeneity within lysosome‐related transcriptional programs. The senescence/inflammation‐enriched subtype represents a microenvironment dominated by senescence‐associated and inflammatory processes, whereas the metabolism‐enriched subtype is primarily associated with metabolic and circadian regulation. These subtypes should be regarded as distinct molecular phenotypes rather than necessarily representing sequential stages of disease progression.

To characterize the immune microenvironment associated with lysosome‐related molecular phenotypes, we compared the ssGSEA enrichment scores of 28 immune‐cell signatures between lysosome_1 and lysosome_2 groups. After Benjamini–Hochberg correction for multiple comparisons, 22 of the 28 signatures remained significantly different between the two groups at an FDR threshold of *q* < 0.05 (Figure 1F, Table S2). Among these, activated B cells, activated CD4 T cells, activated CD8 T cells, activated dendritic cells, CD56^dim^ natural killer cells, central memory CD4 and CD8 T cells, effector memory CD8 T cells, gamma delta T cells, immature B cells, immature dendritic cells, macrophages, mast cells, MDSCs, natural killer cells, natural killer T cells, regulatory T cells, T follicular helper cells, type 1 T helper cells, and type 17 T helper cells also remained significant after the more conservative Bonferroni correction. CD56^bright^ natural killer cells and monocytes remained significant after FDR correction but did not meet the Bonferroni‐adjusted significance threshold. In contrast, effector memory CD4 T cells, eosinophils, memory B cells, neutrophils, plasmacytoid dendritic cells, and type 2 T helper cells were not significant after FDR correction. These findings suggest broad alterations in immune‐related transcriptional signatures associated lysosome. However, because ssGSEA scores reflect relative gene‐set enrichment rather than absolute immune‐cell proportions, these results should be regarded as exploratory evidence of immune microenvironment differences.

### 3.2 Single‐Cell Sequencing Revealed a Positive Association Between NPC Senescence and the Lysosomal Gene‐Signature Score During IVDD

**FIGURE 2:**
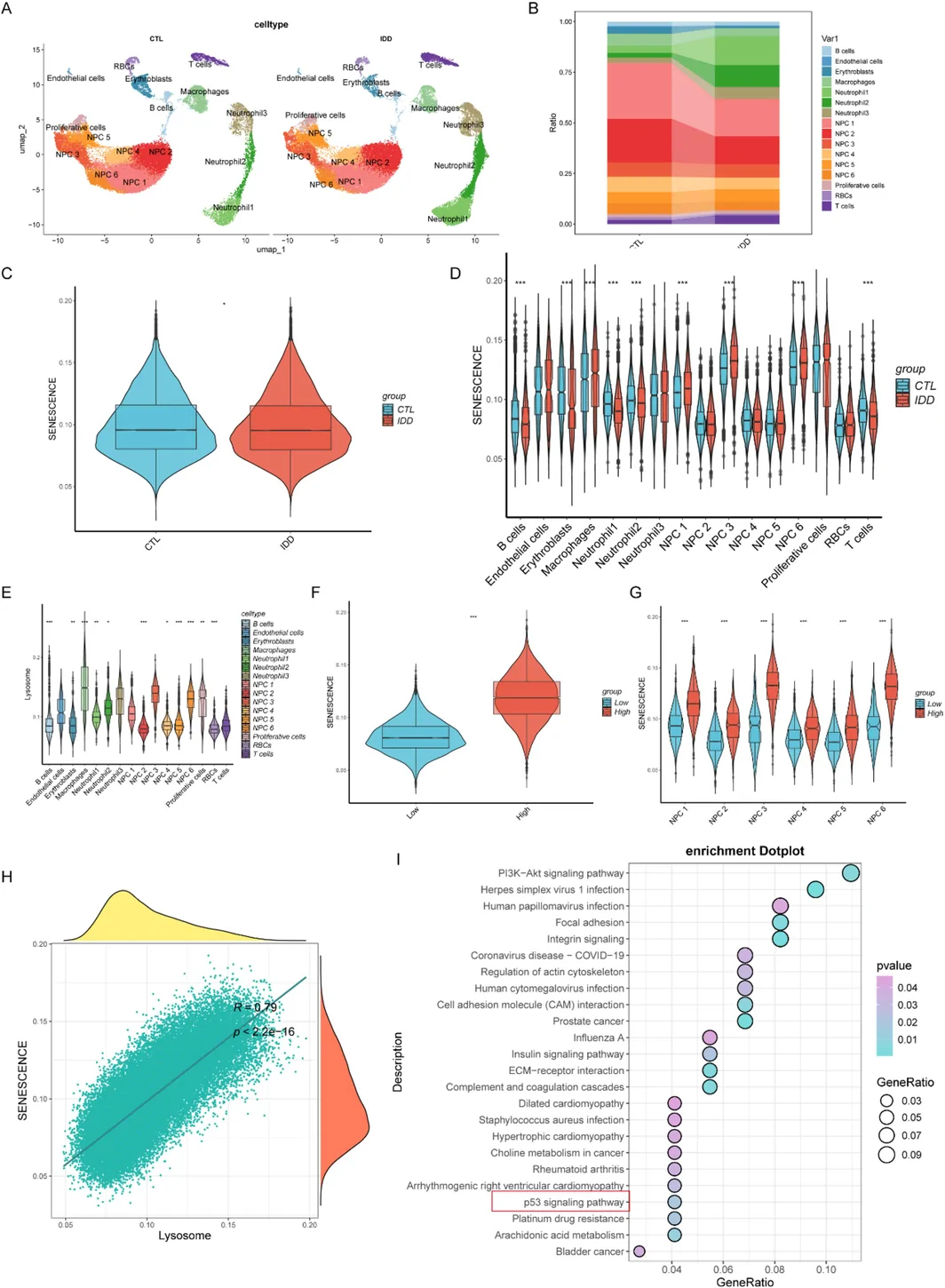
Single‐cell transcriptomic analysis of IVD tissues from IVDD and control groups. (A) UMAP visualization of single‐cell RNA‐sequencing data from human IVD tissues (GSE153066), identifying 16 annotated cell clusters. Each dot represents one cell. (B) Proportions of annotated cell types in the control and IVDD groups. (C) AUCell‐derived senescence scores in control and IVDD samples. (D) Senescence scores across NPC subclusters, stratified by disease status. (E) Lysosomal gene‐signature scores across NPC subclusters. (F) Senescence scores in cells with high versus low lysosomal gene‐signature scores. (G) Senescence scores within each NPC subcluster, stratified by high or low lysosomal gene‐signature scores using the median as the cutoff. (H) Scatter plot showing the association between the lysosomal gene‐signature score and the senescence score in NPCs. The unadjusted correlation coefficient was *r* = 0.79. To control for differences in global transcriptional activity, partial correlation analysis was subsequently performed using nCount_RNA as a covariate. The association remained significant after adjustment (partial *r* = 0.422, permutation *p* < 0.001). (I) KEGG analysis of NPC subclusters based on lysosome gene signature score. Abbreviations: IVDD, intervertebral disc degeneration; KEGG, Kyoto Encyclopedia of Genes and Genomes; NPC, nucleus pulposus cell; UMAP, Uniform Manifold Approximation and Projection.

Previous studies have established that both cellular senescence and lysosomal function play critical roles in the initiation and progression of IVDD [28, 29]. Building on these findings, we further investigated the relationship between senescence and lysosome function. Single‐cell RNA sequencing data from human IVD tissue were obtained from the Gene Expression Omnibus (GEO) database (accession GSE153066) and re‐analyzed, identifying a total of 16 major cell clusters (Figure 2A). Comparative analysis of cell proportions revealed a significant increase in macrophages, neutrophils, and T cells in IVDD patients compared with controls, alongside a decrease in NPCs, particularly the NPC1, NPC2, and NPC4 subpopulations (Figure 2B). Expression profiling indicated that NPC1 and NPC2 were characterized by markers such as KLF4, CNMD, CLU, and CHI3L2, suggesting a healthy NPC phenotype (Figure S1). In contrast, NPC3 and NPC6 exhibited elevated expression of COL3A1, MMP2, and COL6A1, indicative of a degenerated NPC state (Figure S1). Assessment of senescence scores across all NPCs showed significantly higher scores in the IVDD group (Figure 2C). Subcluster analysis revealed that NPC3 and NPC6 exhibited the highest senescence scores among NPC clusters (Figure 2D), which were further elevated in IVDD samples.

Lysosome‐related transcriptional enrichment was evaluated across cell clusters using an AUCell‐derived lysosomal gene‐signature score. This score represents relative LRG expression. NPC3 and NPC6 exhibited higher lysosomal gene‐signature scores than other NPC subclusters (Figure 2E). Stratification of NPCs according to the lysosomal gene‐signature score showed that cells with higher lysosomal transcriptional scores also showed higher senescence scores (Figure 2F). This trend was consistent across all NPC subclusters, with higher lysosome scores correlating with increased senescence (Figure 2G). Notably, this association suggests coordinated activation of lysosome‐related transcriptional programs in senescent or stressed NPCs but does not indicate enhanced lysosomal degradation or establish causal directionality. Initial correlation analysis revealed a strong positive association between the lysosomal gene‐signature score and the senescence score in NPCs (*R* = 0.79, *p* < 2.2 × 10^−16^) (Figure 2H). Because both scores were derived from scRNA‐seq data, we further examined whether this association was influenced by differences in global transcriptional activity. After controlling for nCount_RNA, representing the total UMI count per cell, the association remained significant, with a partial correlation coefficient of *R* = 0.422. In addition, a 2000‐iteration random permutation test showed that the observed partial correlation was located outside the empirical null distribution, with a permutation *p* = 0.0005 (Figure S2). These findings indicate that the association between lysosome‐related and senescence‐related transcriptional programs was not solely attributable to variations in overall cellular transcriptional output. KEGG pathway analysis of NPCs based on lysosome gene signature score further identified several differentially activated pathways associated with cellular senescence, including the p53 signaling pathway, ECM–receptor interaction, PI3K–AKT signaling pathway, and rheumatoid arthritis (Figure 2I).

### 3.3 Identification of DEGs and Enrichment Analysis Based on the Integrated Bulk RNA From Human IVD Tissue

**FIGURE 3:**
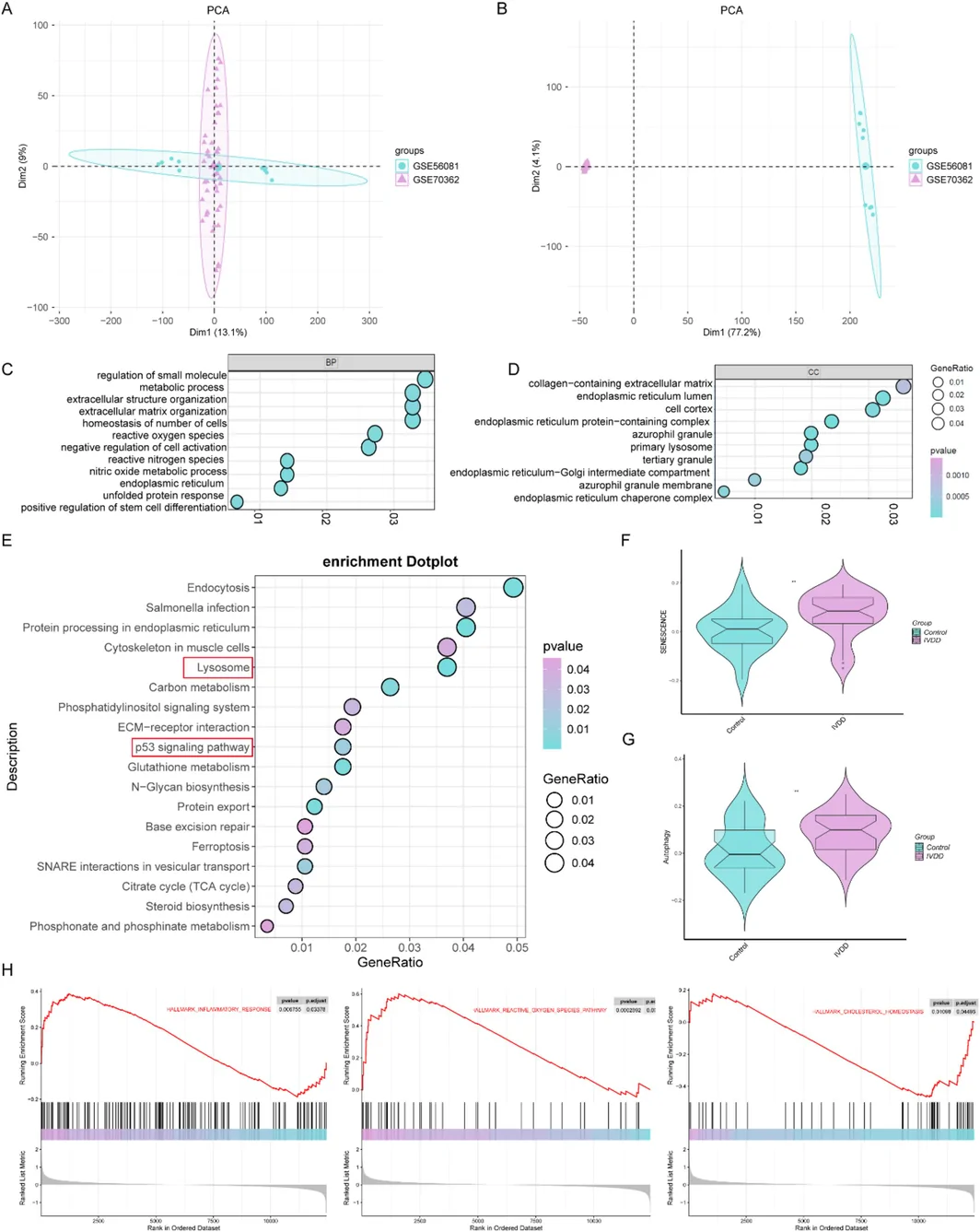
Different expression analysis and function enrichment analysis in normal and degenerated human IVD tissue. (A) PCA of the combined dataset before batch correction. (B) PCA after batch correction. (C) GO biological process enrichment analysis of genes upregulated in IVDD. (D) GO cellular component enrichment analysis of genes upregulated in IVDD. (E) KEGG pathway enrichment analysis of genes upregulated in IVDD. (F) Senescence‐related gene‐signature scores in control and IVDD groups. (G) Autophagy‐related gene‐signature scores in control and IVDD groups. (H) GSEA of inflammatory response, reactive oxygen species, and cholesterol homeostasis pathways in IVDD versus control samples. NES and FDR *q* values are shown. Abbreviations: FDR, false discovery rate; GO, Gene Ontology; GSEA, gene set enrichment analysis; IVDD, intervertebral disc degeneration; KEGG, Kyoto Encyclopedia of Genes and Genomes; NES, normalized enrichment score; PCA, principal component analysis.

To further investigate the regulatory role of lysosomal function in IVDD, we first performed normalization and batch‐effect correction on the datasets GSE56081 and GSE70362. Figure 3A showed the PCA plot of the combined dataset before batch correction, and Figure 3B demonstrated the PCA plot of the combined dataset after batch correction, with samples colored by dataset origin (GSE56081 vs. GSE70362). Samples from the two datasets are well intermingled, indicating successful removal of batch‐related variation (Figure 3A,B). Using the “limma” package, we identified DEGs between healthy control and IVDD samples, applying a significance threshold of *p* < 0.05. A total of 1189 DEGs were detected, comprising 563 upregulated and 626 downregulated genes, as visualized in the volcano plot (Figure S3). To elucidate the underlying molecular mechanisms and functional alterations in IVDD, GO and KEGG pathway enrichment analyses were conducted separately on upregulated and downregulated gene sets. Among the upregulated genes, BPs prominently enriched included extracellular structure organization, reactive oxygen species metabolism, and positive regulation of stem cell differentiation (Figure 3C). In terms of CCs, significant enrichment was observed for collagen‐containing extracellular matrix, endoplasmic reticulum lumen, and primary lysosome (Figure 3D). KEGG pathway analysis further revealed strong associations with lysosome‐related pathways, ECM‐receptor interaction, p53 signaling, ferroptosis, and the TCA cycle (Figure 3E).

Cellular senescence was notably elevated in the IVDD group, as indicated by senescence scoring (Figure 3F). Consistent with the increased lysosome‐related transcriptional enrichment observed in degenerated NPCs, the autophagy‐related gene‐signature score was also significantly elevated in IVDD samples (Figure 3G). This score reflects transcriptional enrichment of autophagy‐associated genes rather than direct measurement of autophagic degradation. Additionally, gene set enrichment analysis (GSEA) was employed to evaluate pathway activation related to inflammatory response, reactive oxygen species (ROS), and cholesterol homeostasis in degenerative NP tissue. The results demonstrated marked activation of inflammatory and ROS‐associated pathways during IVDD progression (Figure 3H).

### 3.4 Construction of Weighted Coexpression Network and Identification of Critical Modules

**FIGURE 4:**
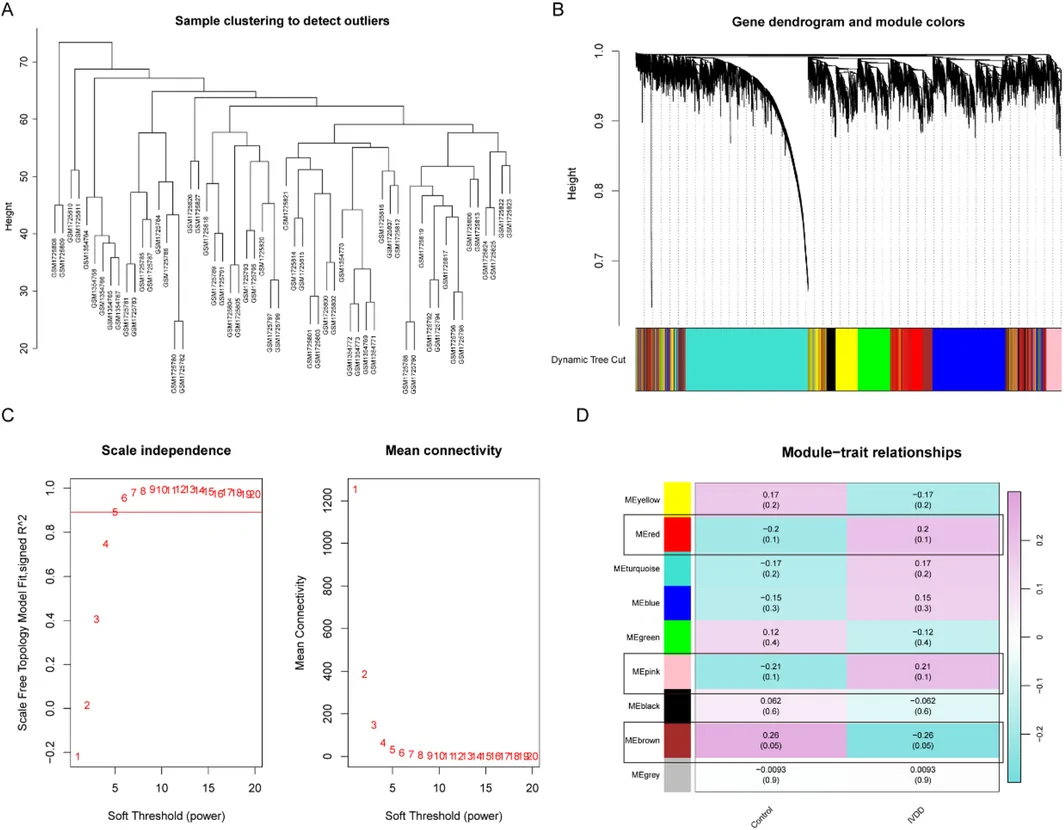
Construction of weighted coexpression network and identification of critical modules. (A) Cluster dendrogram of IVDD. (B) Cluster tree dendrogram of coexpression modules (soft‐threshold power = 5, minimum module size = 50). Different colors represent distinct coexpression modules. (C) The selection of soft threshold power. (D) Heatmap of the correlation between gene modules and clinical traits of IVDD. Abbreviations: IVDD, intervertebral disc degeneration; WGCNA, weighted gene coexpression network analysis.

Utilizing the WGCNA package in conjunction with clinical data, we constructed a coexpression network based on the top 25% most variable genes (Figure 4A). Sample clustering showed robust grouping with no apparent outliers. Gene modules were defined by average linkage hierarchical clustering, each containing a minimum of 50 genes (Figure 4B). The optimal soft‐thresholding power for achieving a scale‐free topology was determined to be 5 (scale‐free *R* ^2^ = 0.85) (Figure 4C). Following the merging of closely related modules, nine distinct coexpression modules were ultimately identified (Figure 4D). Correlation analysis revealed that the red (Cor = −0.20, *p* = 0.10) and pink (Cor = −0.21, *p* = 0.10) modules exhibited negative associations with IVDD, suggesting a potential protective role in disease progression. In contrast, the purple module (Cor = 0.23, *p* = 2.9 × 10^−6^) and brown (Cor = 0.26, *p* = 0.05) module showed a positive correlation with IVDD (Figure 4D), indicating its likely involvement in promoting IVDD pathogenesis. Hub genes from both the positively and negatively correlated modules were subsequently selected for further analysis.

### 3.5 Identification of LRGs Based on Machine Learning Algorithms

**FIGURE 5:**
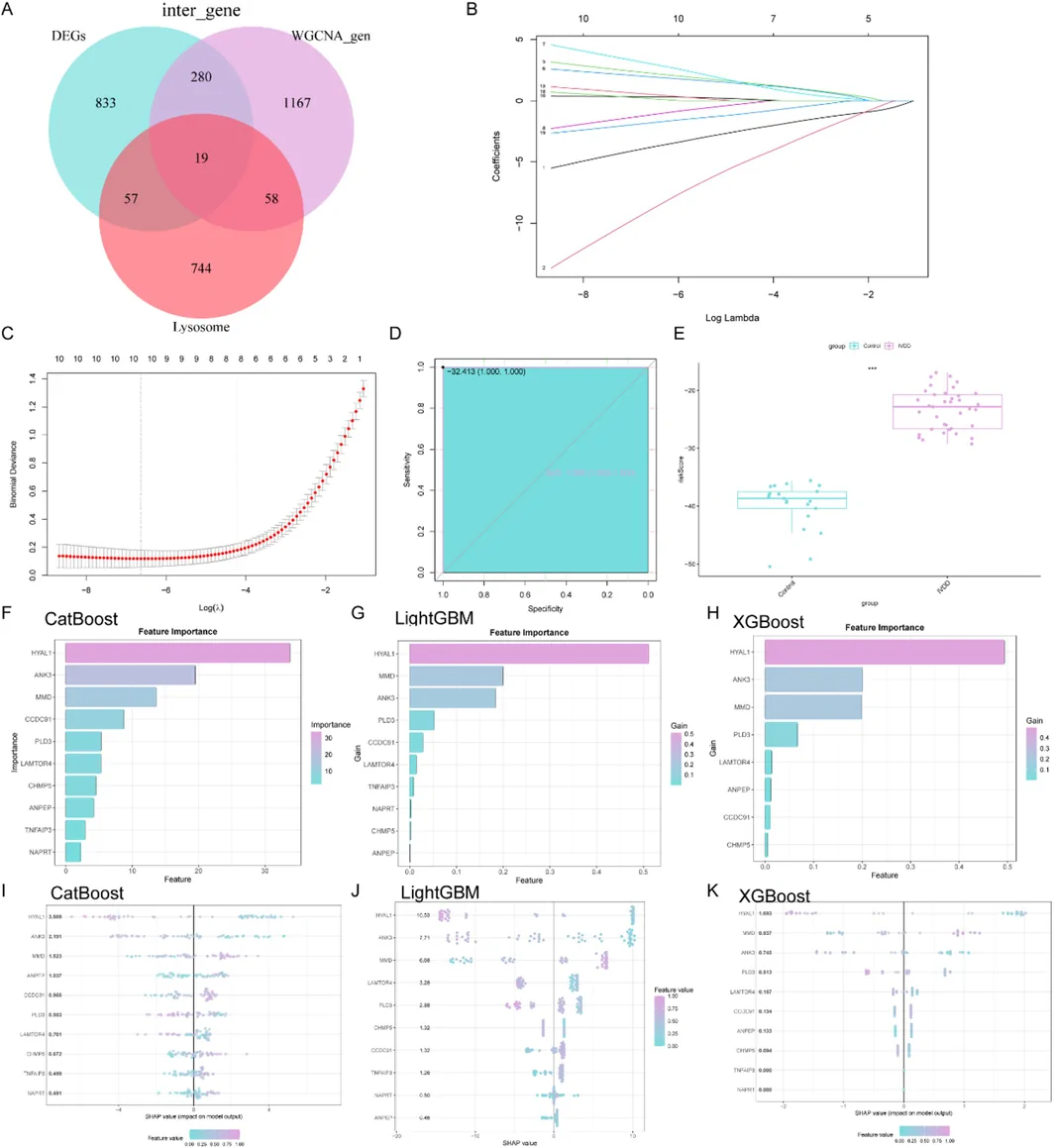
Identification of hub lysosome‐related genes (LRGs) using machine learning algorithms. (A) Venn diagram showing the overlap among DEGs, WGCNA module genes, and LRGs. (B) LASSO coefficient profiles of the 19 candidate genes. (C) LASSO cross‐validation curve used to select the optimal lambda and 10 candidate genes. (D) ROC curve of the LASSO model (AUC = 1.000; *n* = 58). (E) Risk scores in the control and IVDD groups. Data are presented as mean ± SD; significance was assessed using an unpaired Student's *t*‐test. (F–H) SHAP summary bar plots showing feature importance in the CatBoost, LightGBM, and XGBoost models. (I–K) SHAP swarm plots showing the direction and magnitude of each gene's contribution to model predictions. Abbreviations: AUC, area under the curve; DEG, differentially expressed gene; LASSO, least absolute shrinkage and selection operator; LRG, lysosome‑related gene; ROC, receiver operating characteristic; SHAP, SHapley Additive exPlanations; WGCNA, weighted gene coexpression network analysis.

To refine the LRGs, we identified 19 genes via Venn diagrams (Figure 5A). LASSO regression was subsequently employed to further eliminate redundancy, resulting in the selection of 10 potential genes (Figure 5B,C). Furthermore, the LASSO machine learning model was assessed using ROC analysis, with an area under the curve (AUC) value of 1.000 (Figure 5D). Moreover, the risk score for IVDD was found to be significantly higher than that of the control group, suggesting that these genes have potential as diagnostic markers for IVDD (Figure 5E). Further, we used CatBoost, LightGBM, and XGBoost to construct machine learning models for the hub genes. The SHAP summary plot ranking the importance of the feature variables showed that HYAL1, ANK3, and MMD were the top three genes with the highest multimodel contribution (Figure 5F–H). The swarm plot is used to show the distribution and direction of the contribution of each characterized gene to the model prediction, from which we can find that higher HYAL1 and ANK3 expression is associated with lower IVDD incidence, in contrast to higher MMD expression values and positive SHAP values, which have a stronger impact on the prediction of IVDD incidence (Figure 5I–K). Collectively, we deduced that these three genes may be potential candidate genes associated with IVDD.

### 3.6 Construction of a Predictive Nomogram and Validation of LRGs for IVDD

**FIGURE 6:**
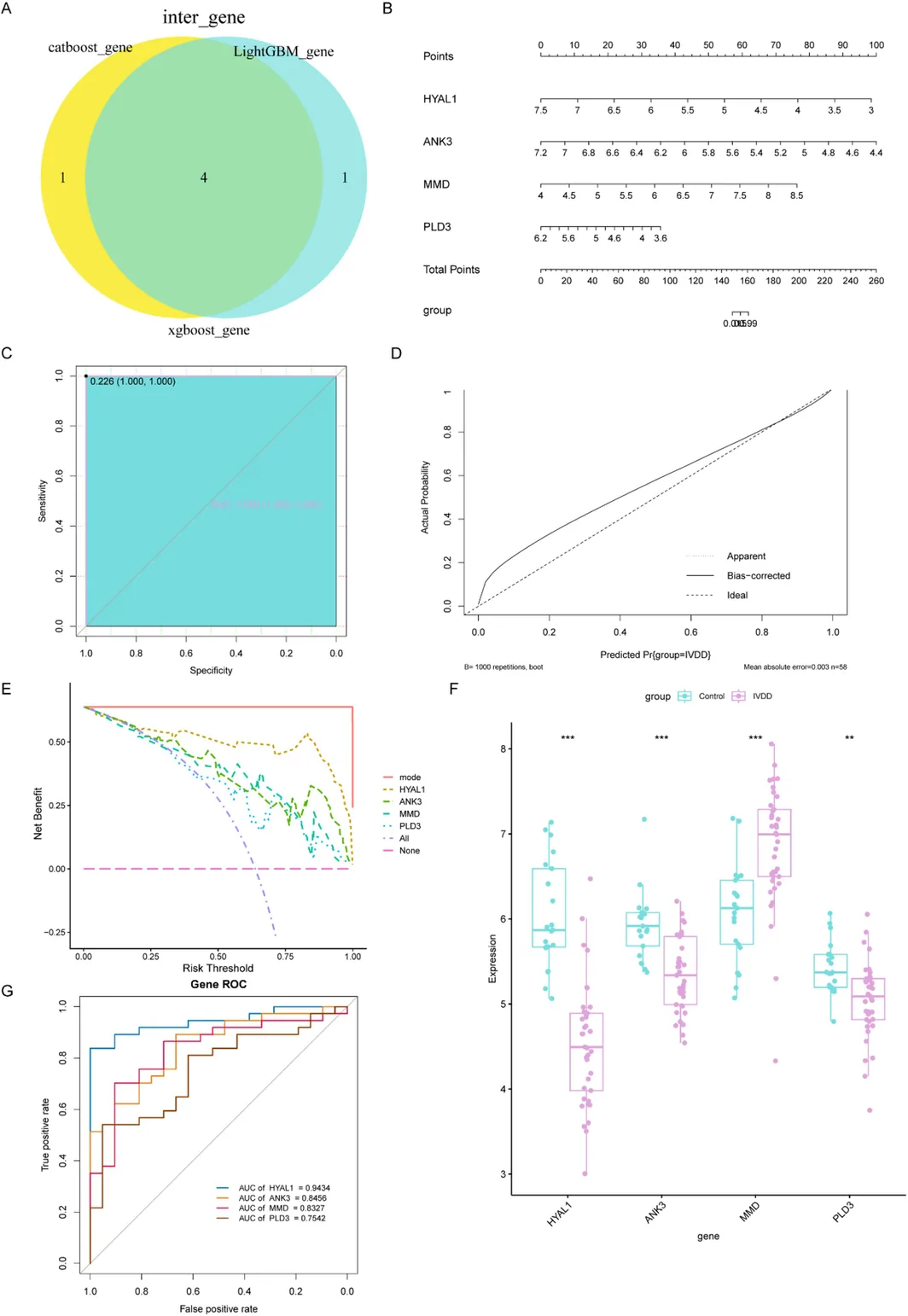
Construction of a predictive nomogram and validation of hub LRGs for IVDD. (A) Venn diagram showing the overlap among the top five genes identified by the CatBoost, LightGBM, and XGBoost models, yielding four hub genes: HYAL1, MMD, PLD3, and ANK3. (B) Nomogram constructed using the four hub genes to estimate IVDD risk. (C) ROC curve of the nomogram model (AUC = 1.000). (D) Calibration curve comparing predicted and observed IVDD probabilities. (E) Decision curve analysis of the nomogram across different threshold probabilities. (F) Expression levels of the four hub genes in control and IVDD groups. Data are presented as mean ± SD; significance was assessed using an unpaired Student's *t*‐test. (G) ROC curves for the individual hub genes. Abbreviations: AUC, area under the curve; DCA, decision curve analysis; IVDD, intervertebral disc degeneration; ROC, receiver operating characteristic.

Machine learning models based on CatBoost, LightGBM, and XGBoost demonstrated strong apparent discrimination in the analyzed cohort for hub genes associated with IVDD. To identify the most reliable candidates, the top five hub genes from each model were selected and their intersection was determined, yielding four final hub genes (Figure 6A). To further evaluate the clinical relevance of these four hub genes, a nomogram prediction model was constructed (Figure 6B). The model achieved an area under the ROC curve (AUC) of 1.000, indicating excellent predictive performance (Figure 6C). The calibration curve demonstrated high consistency between predicted and observed outcomes (Figure 6D). Additionally, DCA was performed for the integrated model of the four genes (Figure 6E). In the training cohort, the expression of MMD was significantly upregulated in the IVDD group, while the expression levels of HYAL1, ANK3, and PLD3 were notably lower compared with the control group (Figure 6F). ROC analysis of these hub genes yielded AUC values of 0.9434, 0.8456, 0.8327, and 0.7542, respectively, highlighting their potential as promising biomarkers for IVDD (Figure 6G).

To investigate the effect of hub genes on NPC homeostasis, we selected PLD3 to perform bidirectional gain‐ and loss‐of‐function experiments because of its lysosomal localization, its downregulation in IVDD tissues, and its strong association with lysosome‐ and senescence‐related transcriptional signatures in our integrated analyses. Under H_2_O_2_‐induced oxidative stress, PLD3 knockdown further reduced LysoTracker fluorescence and cathepsin B activity, demonstrating that PLD3 deficiency exacerbated H_2_O_2_‐induced lysosomal dysfunction (Figure S5A–C). PLD3 knockdown also exacerbated the degenerative and senescent phenotype of NPCs, as demonstrated by decreased ACAN expression and increased MMP13 and CDKN1A expression (Figure S5D). Conversely, PLD3 overexpression significantly restored LysoTracker fluorescence and cathepsin B activity and reversed the corresponding degeneration‐ and senescence‐associated gene‐expression changes. Therefore, PLD3 contributes to the maintenance of lysosomal homeostasis and protects NPCs against oxidative stress‐induced degeneration and senescence.

### 3.7 Immune Characteristics and Interaction Function of LRGs

The progression of IVDD leads to elevated levels of inflammatory cytokines within IVD tissues, promoting immune cell infiltration and further amplifying the inflammatory cascade.

**FIGURE 7:**
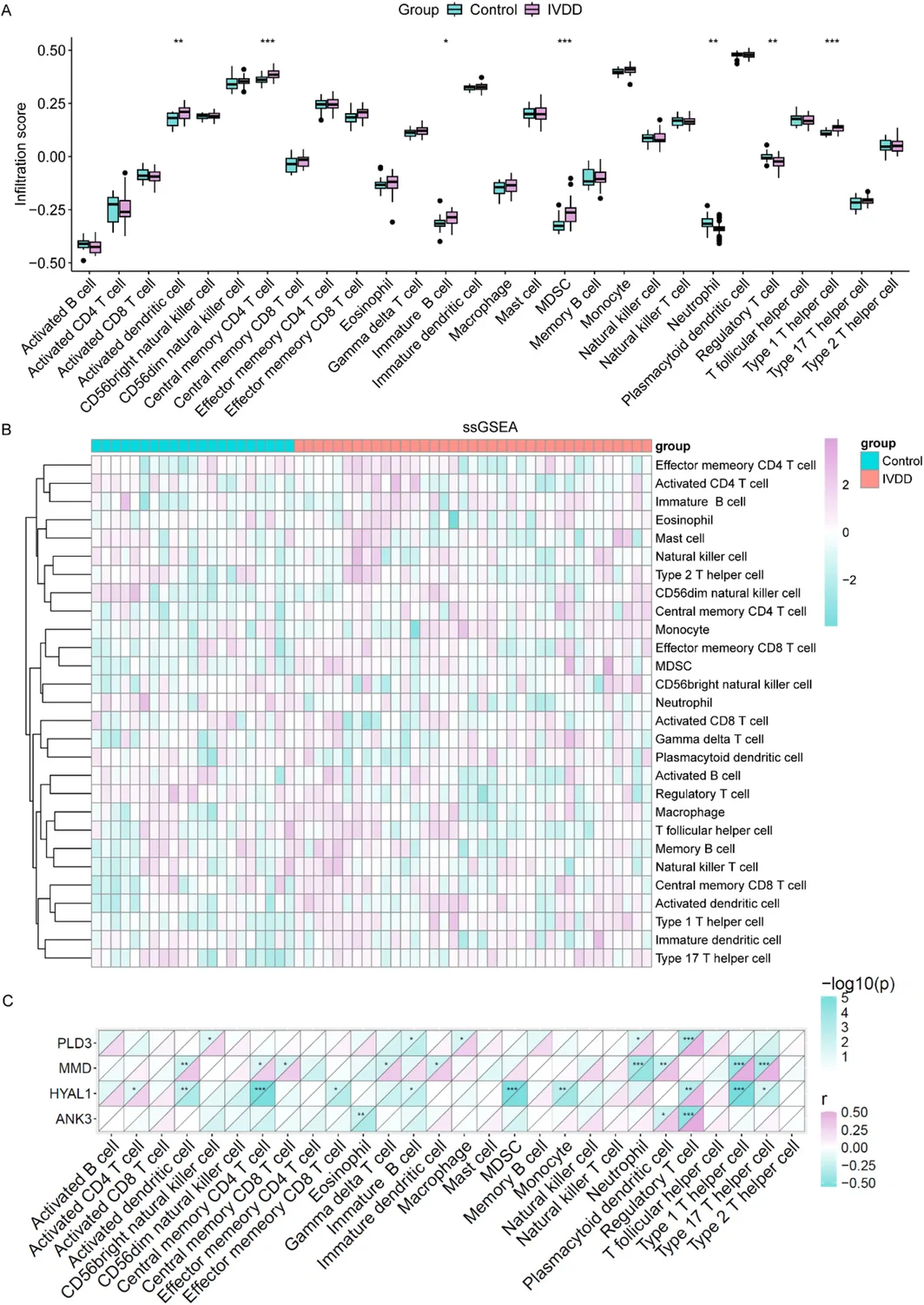
Exploratory analysis of immune‐cell transcriptional signatures in intervertebral disc degeneration. (A) Violin plots comparing the relative abundance of 28 immune cell types between control and IVDD groups, estimated by single‐sample gene set enrichment analysis (ssGSEA). (B) Heatmap showing ssGSEA enrichment scores for 28 immune cell types in individual control and IVDD samples. Rows represent immune cell types, columns represent samples. Color scale indicates enrichment score. (C) Correlation between HYAL1, MMD, PLD3, and ANK3 expression and immune cells in the IVDD group. Pearson correlation coefficients are shown, with color indicating correlation strength. Red: positive correlation, blue: negative correlation. Abbreviations: FDR, false discovery rate; IVDD, intervertebral disc degeneration; MDSC, myeloid‐derived suppressor cell; ssGSEA, single‐sample gene set enrichment analysis.

Immune infiltration analysis revealed significant differences in several immune‐cell populations between the IVDD and control groups (Figure 7A). After Benjamini–Hochberg correction for multiple comparisons, seven of the 28 immune‐cell signatures remained statistically significant at an FDR‐adjusted *q* value of < 0.05 (Table S3). These signatures included activated dendritic cells, central memory CD4 T cells, immature B cells, myeloid‐derived suppressor cells (MDSCs), neutrophils, regulatory T cells, and type 1 T helper cells. When the more stringent Bonferroni correction was applied, activated dendritic cells, central memory CD4 T cells, MDSCs, and type 1 T helper cells remained statistically significant (Table S3). In contrast, the associations observed for immature B cells, neutrophils, and regulatory T cells did not remain significant after Bonferroni correction. Among all immune‐cell signatures, type 1 T helper cells and MDSCs showed the strongest statistical evidence of differences according to IVDD status (Table S3). Collectively, these findings indicate that IVDD is associated with alterations in specific innate and adaptive immune‐cell populations.

Correlation analysis between the expression of the four hub genes (HYAL1, MMD, PLD3, and ANK3) and immune cell proportions revealed distinct immune‐gene associations in IVDD (Figure 7C). PLD3 expression was negatively correlated with CD56^dim^ natural killer cells, immature B cells, macrophages, neutrophils, and regulatory T cells. MMD expression showed negative correlations with activated dendritic cells, central memory CD4^+^ and CD8^+^ T cells, gamma delta T cells, immature dendritic cells, neutrophils, plasmacytoid dendritic cells, type 1 T helper cells, and type 17 T helper cells. HYAL1 expression was negatively associated with activated CD4^+^ T cells, activated dendritic cells, central memory CD4^+^ T cells, effector memory CD8^+^ T cells, immature B cells, MDSCs, monocytes, regulatory T cells, type 1 T helper cells, and type 17 T helper cells. ANK3 expression exhibited negative correlations with eosinophils, plasmacytoid dendritic cells, and regulatory T cells (Figure 7C). Importantly, these correlations represent associative relationships rather than causal links, and the directionality, whether hub gene expression influences immune cell recruitment or immune activity modulates gene expression, cannot be determined from these data. Nevertheless, these findings generate testable hypotheses regarding the potential immunomodulatory roles of these genes in the IVDD microenvironment. For instance, the strong negative correlations of HYAL1 with multiple T‐cell subtypes suggest that HYAL1 downregulation in degenerating NP tissue may create a permissive environment for T‐cell infiltration, warranting future functional studies to explore this potential immune regulatory axis.

Subsequently, lysosome‐ and senescence‐related gene sets were retrieved from the GeneCards database2, from which the expression levels of the top 20 genes were extracted. Furthermore, our analysis revealed close associations between the four hub genes and lysosome‐ or senescence‐related genes in the context of IVDD (Figure S4).

These findings highlight the potential importance of the four key genes, HYAL1, MMD, PLD3, and ANK3, in regulating immune responses, lysosome function, and cellular senescence associated with IVDD, suggesting a comparable immunological profile shared by both conditions.

### 3.8 Hub Gene Function and Histological Analysis Confirmed the Relationship Between Lysosome Dysfunction and IVDD

**FIGURE 8:**
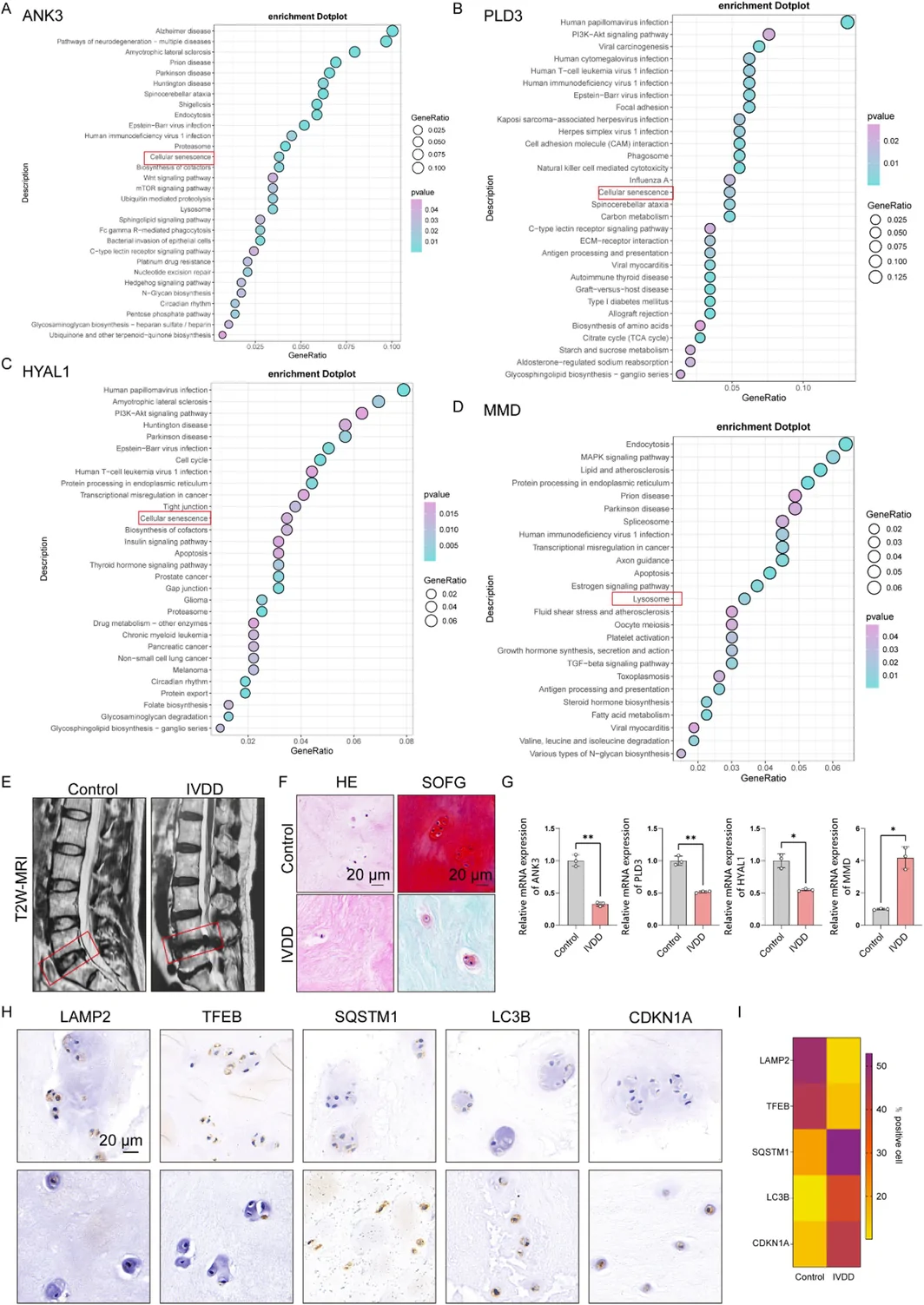
Hub gene functional analysis and histological validation in human IVD tissues. (A–D) KEGG analysis based on the hub genes. (E) Representative T2‐weighted MRI of lumbar intervertebral disc. (F) Representative images of H&E and SOFG staining for human IVD tissue. Scale bar = 20 μm. (G) The results of qRT‐PCR of human IVD tissue in control and IVDD group, respectively (*n* = 3). (H,I) Representative images and quantitation of IHC staining for LAMP2, TFEB, SQSTM1, LC3B, and CDKN1A in human IVD tissue. Scale bar = 20 μm. *p < 0.05, **p < 0.01, ***p < 0.001 and ****p < 0.0001. Abbreviations: H&E, hematoxylin and eosin; IHC, immunohistochemistry; IVDD, intervertebral disc degeneration; KEGG, Kyoto Encyclopedia of Genes and Genomes; MRI, magnetic resonance imaging; qRT‑PCR, quantitative reverse transcription polymerase chain reaction; SOFG, safranin O/fast green.

To explore the regulatory functions of the four hub genes in IVDD, KEGG pathway enrichment analysis was performed. The results revealed distinct pathway associations for each gene: ANK3 was significantly enriched in pathways related to cellular senescence, Wnt signaling, mTOR signaling, lysosome function, and circadian rhythm (Figure 8A). PLD3 was associated with focal adhesion, cellular senescence, carbon metabolism, and the TCA cycle (Figure 8B). HYAL1 showed enrichment in the PI3K‐Akt signaling pathway, cell cycle regulation, cellular senescence, and apoptosis (Figure 8C). MMD was linked to the MAPK signaling pathway, lysosome function, apoptosis, and TGF‐β signaling (Figure 8D).

Furthermore, human IVD tissues were collected and classified into a control group (Pfirrmann grade II) and an IVDD group (Pfirrmann grades III, IV, and V) based on preoperative radiological assessment (Figure 8E). Histological evaluation using H&E and SO/FG staining confirmed the reliability of the radiological grading, showing reduced proteoglycan content in degenerated IVD tissues (Figure 8F). Analysis of the four hub genes in human IVD tissues revealed that degenerated tissues exhibited significantly higher expression of MMD and lower expression of ANK3, PLD3, and HYAL1, consistent with the bulk RNA sequencing results (Figure 8G). Given the critical role of lysosomes in autophagy, we further examined the expression of autophagy‐ and senescence‐related proteins. Immunohistochemical analysis showed that degenerated NP tissues exhibited decreased expression of LAMP2 and TFEB compared with control NP tissues, indicating impaired lysosomal biogenesis during IVDD progression (Figure 8H,I). Additionally, increased expression of SQSTM1 and LC3B, two markers of autophagic degradation, was observed in degenerated NP tissues, suggesting a defect in autophagic degradation (Figure 8H,I). Notably, elevated expression of CDKN1A was also detected in the IVDD group (Figure 8H,I).

Collectively, these findings suggest that lysosomal dysfunction and impaired autophagic degradation are closely associated with cellular senescence and the pathogenesis of IVDD.

### 3.9 Lithocholic Acid (LA) Partially Improves Lysosomal Functional Measures and Attenuates Degeneration‐Associated Changes In Vitro and In Vivo

**FIGURE 9:**
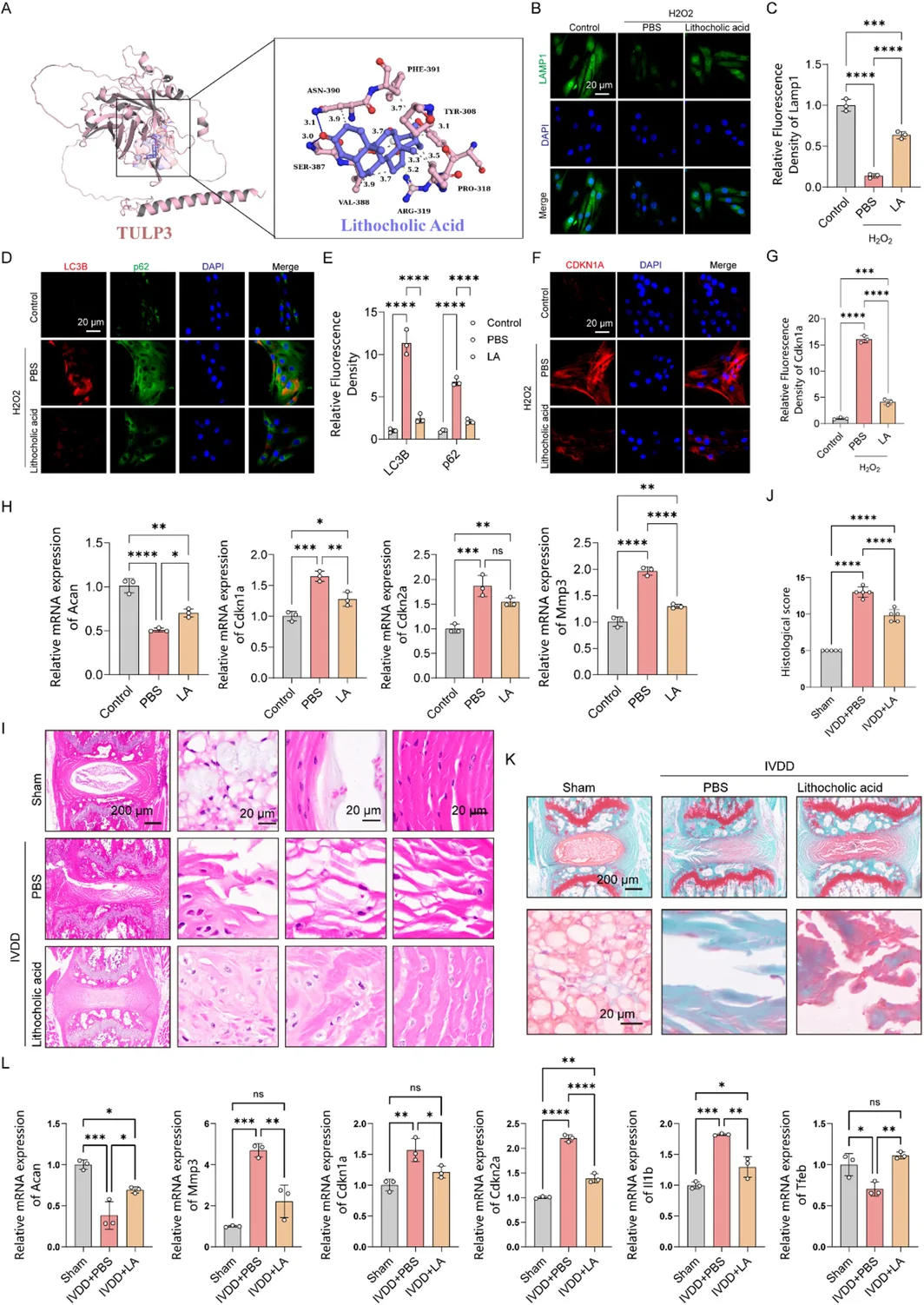
Lithocholic acid improves lysosomal homeostasis and attenuates autophagy‐, senescence‐, and degeneration‐associated changes in experimental models of IVDD. (A) Molecular docking of lithocholic acid with TULP3. B, (C) Representative LAMP1 immunofluorescence images and quantification in NPCs treated with H_2_O_2_ with or without LA for 24 h (*n* = 3). (D,E) Representative LC3B and p62 immunofluorescence images and quantification (*n* = 3). (F,G) Representative CDKN1A immunofluorescence images and quantification (*n* = 3). (H) RT‐qPCR analysis of degeneration‐ and senescence‐related genes in NPCs (*n* = 3). (I,J) Representative H&E‐stained rat IVD sections and histological scores in the sham, IVDD, and LA groups (*n* = 5). (K) Representative safranin O/fast green‐stained rat IVD sections. (L) RT‐qPCR analysis of rat IVD tissues (*n* = 3). Scale bars = 20 μm for immunofluorescence images and 20/200 μm for histological images. *p < 0.05, **p < 0.01, ***p < 0.001, and ****p < 0.0001. Abbreviations: H&E, hematoxylin and eosin; IVDD, intervertebral disc degeneration; LA, lithocholic acid; NPC, nucleus pulposus cell; RT‐qPCR, quantitative reverse transcription polymerase chain reaction; SOFG, safranin O/fast green.

To investigate whether restoring lysosomal function could mitigate cellular senescence and IVDD, we conducted a series of in vitro and in vivo experiments. LA has been reported to bind TULP3, thereby enhancing the activity of the deacetylase Sirtuins. This leads to deacetylation and inhibition of V1E1, a key subunit of the lysosomal proton pump v‐ATPase, supporting lysosomal function [30]. Based on this reported mechanism, we selected LA as a potential agent to improve lysosomal homeostasis and evaluated its effects using direct lysosomal functional assays. Molecular docking predicted a favorable binding pose between LA and TULP3, with a docking score of −8.1 kcal/mol (Figure 9A). As illustrated in Figure 9A, the ligand achieves stable binding with the target protein through hydrophobic interactions, hydrogen bonds, and a salt bridge: residues 308A‐TYR (3.15 Å), 318A‐PRO (3.33 Å), 388A‐VAL (3.75 Å), 391A‐PHE (3.69 Å), 366A‐PRO (3.69 Å), 319A‐ARG (3.73 Å), 388A‐VAL (3.87 Å), and 390A‐ASN (3.94 Å) form an extensive hydrophobic interaction interface. Simultaneously, 387A‐SER (3.04 Å) and 390A‐ASN (3.12 Å) form critical hydrogen bonds with the ligand. Furthermore, 319A‐ARG (5.24 Å) forms a salt bridge interaction with the carboxylate group of the ligand. Together, these interactions enhance the binding affinity and specificity of the complex.

First, we evaluated the cytotoxic effects of LA on rat NPCs using a concentration range of 0–100 μM. Among the tested concentrations, 10‐μM LA produced the highest relative cell‐viability signal without an apparent reduction in viability (Figure S6). Therefore, this concentration was selected for the subsequent experiments. To distinguish lysosome‐related transcriptional changes from actual lysosomal functional status, we directly examined acidic lysosomal compartments and lysosomal proteolytic activity in H_2_O_2_‐induced senescent NPCs. LysoTracker staining showed abundant punctate red fluorescence in control NPCs, whereas H_2_O_2_ treatment markedly reduced the LysoTracker signal (Figure S7A). Quantitative analysis confirmed a significant decrease in relative LysoTracker fluorescence intensity following H_2_O_2_ exposure (Figure S7B), indicating a reduction in acidic lysosomal compartments. Notably, LA treatment significantly restored LysoTracker fluorescence, although the signal remained slightly lower than that in the control group. Consistent with the LysoTracker findings, fluorometric analysis demonstrated that cathepsin B activity was significantly reduced in H_2_O_2_‐treated NPCs compared with control cells (Figure S7C). LA administration partially restored cathepsin B activity, indicating an improvement in lysosomal proteolytic capacity. Together, these direct functional measurements demonstrate that H_2_O_2_‐induced NPC senescence is accompanied by impaired maintenance of acidic lysosomal compartments and decreased lysosomal protease activity, both of which can be partially alleviated by LA treatment.

We next examined lysosome‐, autophagy‐, and senescence‐associated markers. H_2_O_2_‐induced senescent NPCs exhibited reduced LAMP1 expression together with LC3B and SQSTM1/p62 accumulation, which was consistent with altered lysosomal homeostasis and impaired autophagic degradation (Figure 9B–E). Increased CDKN1A immunofluorescence staining further confirmed successful induction of cellular senescence (Figure 9F,G). Notably, LA treatment partially attenuated these alterations (Figure 9B–G). Furthermore, the results of RT‐qPCR indicated that the administration of LA significantly alleviated NPC senescence and degeneration (Figure 9H).

To extend these findings beyond in vitro conditions, we administered LA in a rat IVDD model to evaluate its therapeutic potential in vivo. In our study, we chose local administration for the following reasons: (1) The IVD is an avascular tissue with limited penetration of systemically administered drugs. Thus, local administration was selected as a proof‐of‐concept approach to increase exposure within the avascular disc and potentially limit systemic exposure. (2) This route bypasses first‐pass metabolism and avoids potential off‐target effects associated with systemic exposure to LA, such as its known effects on enterohepatic circulation. (3) Intradiscal injections are already a part of clinical practice for delivering analgesics and other agents to treat discogenic pain, making this a translationally relevant route of administration. At the endpoint of the experiment, we evaluated the IVD tissue histologically. Histological analysis using H&E and safranin O/fast green (SF) staining revealed severe disruption of the boundary between the NP and AF, and the decreased proteoglycan in the IVDD group, which was partially attenuated in LA‐treated discs (Figure 9I–K). RT‐qPCR results showed that IVDD was associated with downregulation of Acan and Tfeb, and upregulation of Mmp3, Cdkn1a, Cdkn2a, and Il1b; these expression changes were significantly attenuated by LA administration (Figure 9L). We further assessed the expression of hub genes in rat IVD tissue. Consistent with findings in human tissue, degenerated discs exhibited downregulation of ANK3, HYAL1, and PLD3, which was partially shifted toward control‐group levels following LA administration. In contrast, MMD expression was elevated in degenerated tissue and was reduced following LA administration (Figure S8).

In summary, these findings suggest that LA partially improves lysosomal functional status and autophagic degradation, attenuates senescence‐associated alterations in H_2_O_2_‐treated NPCs, and reduces degeneration‐associated histological and molecular changes in the rat IVDD model.

## 4 Discussion

Our single‐cell analysis demonstrated that degeneration‐associated NPC subpopulations exhibited both elevated senescence scores and elevated lysosomal gene‐signature scores. Importantly, the latter reflects increased expression of lysosome‐related genes rather than direct enhancement of lysosomal degradative function. Such transcriptional activation may represent an adaptive increase in lysosomal biogenesis or a compensatory response to oxidative stress, substrate accumulation, and impaired proteostasis. Thus, increased lysosomal gene expression can coexist with reduced acidic lysosomal compartments, reduced hydrolase activity, and impaired autophagic degradation. Such compensatory transcriptional activation does not necessarily indicate enhanced degradative capacity. Instead, it often reflects lysosomal overload, membrane permeabilization, and diminished hydrolase activity. Such a state can lead to impaired autophagic degradation, as evidenced by the accumulation of LC3B and p62 in degenerated human NP tissues and H_2_O_2_‐induced senescent NPCs. Further, through integrated multi‐omics data, we first identified distinct lysosome‐related molecular subtypes and prioritized four candidate hub genes (HYAL1, MMD, PLD3, and ANK3). Among these candidates, PLD3 was functionally evaluated using bidirectional knockdown and overexpression experiments. Collectively, these findings suggest that dysregulated lysosomal homeostasis is closely associated with impaired autophagic degradation, cellular senescence, and inflammatory activation during IVDD.

In the present study, we identified two biologically distinct lysosome‐related molecular subtypes of IVDD. Cluster 1, designated the senescence/inflammation‐enriched subtype, was characterized by activation of cellular senescence, cell‐cycle dysregulation, inflammatory pathways, and increased immune‐cell infiltration. Senescent cells can release senescence‐associated secretory phenotype (SASP) factors, including pro‐inflammatory cytokines, chemokines, and matrix‐degrading enzymes, thereby promoting immune recruitment, extracellular‐matrix degradation, and a self‐sustaining degenerative microenvironment [31, 32]. This subtype may therefore represent an inflammation‐ and catabolism‐dominant phenotype of IVDD. In contrast, Cluster 2, designated the metabolism‐enriched subtype, exhibited enrichment of steroid biosynthesis, fatty‐acid biosynthesis, and circadian‐rhythm pathways. These metabolic programs may reflect differences in lipid handling, energy utilization, circadian regulation, and cellular adaptation to stress that contribute to the maintenance of tissue homeostasis [26, 33]. However, because the present data are cross‐sectional, these subtypes should be interpreted as distinct molecular phenotypes rather than sequential stages of IVDD. From a potential clinical perspective, this classification may provide a framework for future patient stratification: the senescence/inflammation‐enriched subtype may be more suitable for therapies targeting senescent cells, inflammatory signaling, or lysosomal dysfunction, whereas the metabolism‐enriched subtype may benefit from strategies aimed at preserving metabolic and circadian homeostasis. Nevertheless, these therapeutic implications remain exploratory and require validation in prospective cohorts with clinical outcome and treatment‐response data.

Furthermore, through single‐cell transcriptomic analysis, we identified specific degeneration‐related NPC subpopulations. NPC3 and NPC6 exhibited concurrent increases in senescence scores and lysosomal gene‐signature scores. This coordinated increase indicates activation of lysosome‐related transcriptional programs in stressed or senescent NPCs but does not demonstrate enhanced lysosomal degradative function. However, during advanced IVDD, chronic oxidative stress and metabolic dysregulation would lead to the abnormal accumulation of indigestible agents within lysosomes, triggering lysosomal membrane permeabilization, leakage of cathepsins, and activation of inflammasome pathways [29, 34]. Such transcriptional activation may represent compensatory lysosomal biogenesis in response to oxidative stress and impaired proteostasis. Consistent with this interpretation, H_2_O_2_‐treated NPCs exhibited reduced LysoTracker fluorescence and cathepsin B activity together with LC3B and SQSTM1/p62 accumulation. Thus, elevated lysosome‐related transcription can coexist with impaired acidic lysosomal compartments, reduced proteolytic capacity, and defective autophagic degradation. In fact, a previous study from Liu et al. also identified that mechanical overloading impaired autophagic degradation in NPCs via lysosomal dysfunction, and that restoring lysosomal function through CHMP4B or TFEB overexpression alleviated IVDD [35]. A recent study by Wu et al. also found that improving chaperone‐mediated autophagy (CMA), a lysosome‐dependent protein degradation pathway, could effectively antagonize autophagy‐dependent ferroptosis and NPC senescence [36]. These results above also align with observations in neurodegenerative diseases, where lysosomal hyperactivity often precedes systemic failure [37, 38, 39].

To further investigate lysosome‐related genes associated with IVDD, we applied machine‐learning approaches to the integrated transcriptomic data and prioritized four candidate hub LRGs (HYAL1, MMD, PLD3, and ANK3). Functional enrichment analyses implicated these genes in critical pathways, such as PI3K‐Akt, mTOR, MAPK, and TGF‐β signaling, all of which converge on autophagy and cellular senescence. Hyaluronan (HA), a major glycosaminoglycan abundantly present in the extracellular matrix, plays essential roles in embryonic cell migration, proliferation, differentiation, and structural support in connective tissues. Prior studies have shown that HYAL1 mutations can lead to a recently characterized lysosomal storage disorder known as mucopolysaccharidosis IX, suggesting a potential role for lysosomes in HA homeostasis maintenance [40]. Therefore, the reduced HYAL1 expression observed in IVDD may be associated with altered hyaluronan turnover and lysosomal homeostasis. Nevertheless, whether HYAL1 deficiency causes intralysosomal hyaluronan accumulation or impaired degradative capacity in NPCs remains to be experimentally determined. PLD3 is a lysosome‐localized phospholipase D family member that contributes to the synthesis of bis(monoacylglycero)phosphate, a lipid involved in lysosomal lipid degradation [41]. PLD3 has also been implicated in mitochondrial DNA degradation, lysosomal nucleotide turnover, cGAS–STING signaling, and amyloid precursor protein metabolism [42]. In the present study, PLD3 knockdown aggravated, whereas PLD3 overexpression attenuated, H_2_O_2_‐induced alterations in lysosomal functional measures and degeneration‐ and senescence‐associated markers. These findings provide experimental support for a role of PLD3 in maintaining lysosomal homeostasis under oxidative stress. However, whether this effect is mediated through altered BMP synthesis, lipid accumulation, or lysosomal membrane integrity remains hypothetical. ANK3 is primarily recognized as a scaffold protein that anchors cytoskeletal components to specialized membrane domains, and previous evidence has linked ANK3‐associated lysosomal dysfunction to late‐onset depressive disorder [43]. Its potential involvement in lysosomal positioning or trafficking in NPCs is therefore plausible but has not been directly examined in this study. Similarly, MMD has been associated with macrophage differentiation and cytokine signaling, raising the possibility that it may influence inflammatory or SASP‐related responses; however, its specific function in resident NPCs remains unclear. Collectively, our data identify HYAL1, MMD, PLD3, and ANK3 as candidate genes associated with lysosomal dysregulation in IVDD. Among them, PLD3 is supported by direct functional experiments, whereas the proposed roles of HYAL1, ANK3, and MMD should be regarded as hypothesis‐generating interpretations requiring gene‐specific mechanistic validation.

After correction for multiple comparisons, most of the observed differences in immune‐cell transcriptional signatures remained statistically significant, supporting broad immune‐associated alterations in IVDD. Notably, our correlation analysis revealed strong negative correlations between these hub genes and specific immune cell populations suggesting their potential regulatory influence on the immune microenvironment within IVD tissue. The observed negative correlations could reflect at least two non‐mutually exclusive scenarios: (1) reduced expression of these genes in degenerating NP cells directly promotes immune cell recruitment through loss of immunomodulatory signals, or (2) infiltrating immune cells and their secreted inflammatory cytokines actively suppress the expression of these genes in resident NP cells. For example, the strong negative correlation between HYAL1 and multiple T‐cell subsets raises the possibility that HYAL1, beyond its canonical role in hyaluronan degradation, may participate in immune regulation within the avascular disc microenvironment. Given that hyaluronan fragments generated by HYAL1 activity can signal through TLR2 and TLR4 to modulate immune responses [44], it is plausible that reduced HYAL1 expression alters the local hyaluronan fragment profile, thereby influencing T‐cell recruitment or activation. While these associations are compelling, the precise mechanistic roles of these genes in NPCs warrant further speculation based on their known MFs. Notably, while our ssGSEA analysis identified most immune cell populations with nominally significant differences between IVDD and control groups, we did not apply multiple testing correction across the 28 comparisons performed. This would increase the risk of Type I errors, and therefore these findings should be interpreted as exploratory rather than definitive. In addition, ssGSEA itself has inherent limitations: the method is sensitive to gene set overlap, as many immune cell signatures share common marker genes, which can lead to collinearity and potential overestimation of certain populations. ssGSEA scores reflect relative enrichment within each sample rather than absolute cell proportions, and results can be influenced by overall tissue cellularity and sample composition.

The most direct translational evidence from our study was provided by the successful intervention with LA. In vitro, LA significantly increased LysoTracker fluorescence and restored cathepsin B activity in H_2_O_2_‐induced senescent NPCs, indicating improved maintenance of acidic lysosomal compartments and lysosomal proteolytic capacity. LA also reduced the accumulation of LC3B and SQSTM1/p62 and attenuated CDKN1A expression, supporting the restoration of autophagic degradation and suppression of cellular senescence. In vivo, LA preserved the structural boundary between the NP and AF and maintained proteoglycan content in the rat IVDD model. At the transcriptional level, LA reduced the expression of catabolic and inflammatory genes, including Mmp3 and Il1b, while increasing the expression of the anabolic marker Acan and the lysosomal biogenesis regulator Tfeb. These findings support pharmacological restoration of lysosomal homeostasis as a potential therapeutic strategy for IVDD, distinct from conventional anti‐inflammatory approaches [30].

There are several limitations in this study. First, the clinical cohort's data were generated from public data, and larger prospective studies are needed to validate the diagnostic and prognostic utility of the LRG‐based subtypes and hub genes. In addition, the machine‐learning and nomogram models were evaluated primarily within the analyzed cohort, and their apparently high performance may reflect overfitting. Independent external validation is required before diagnostic application. Second, the precise mechanistic links between individual hub genes and lysosomal pH regulation require further detailed investigation. Third, while the rat model recapitulates key features of IVDD, interspecies differences necessitate caution in extrapolating results to humans. Fourth, although we controlled for total UMI counts per cell using partial correlation analysis and further validated the association through random permutation testing, residual confounding from other cell‐level factors, including cell‐cycle state, cell subtype composition, and additional technical variation, cannot be completely excluded. Future studies using larger single‐cell datasets and independent cohorts will be needed to further confirm the robustness of this association. Fifth, although Benjamini–Hochberg FDR correction and Bonferroni adjustment were applied to the 28 immune‐cell signature comparisons, the ssGSEA results remain exploratory. ssGSEA scores reflect relative enrichment of predefined transcriptional signatures rather than absolute immune‐cell proportions and may be influenced by gene‐set overlap, tissue cellularity, and sample composition. Future studies using immunohistochemistry, flow cytometry, or spatially resolved approaches are required to validate the inferred immune‐cell alterations. Finally, although 10 μM was selected from an in vitro concentration–response experiment and 3 μL was chosen to reduce injection leakage, the actual LA concentration, spatial distribution, and retention time within the disc were not measured. Consequently, the current dosing regimen should be regarded as an empirically selected proof‐of‐concept regimen rather than an optimized therapeutic dose. Future studies should quantify LA in disc tissues at serial time points using LC–MS/MS or labeled‐tracer approaches and compare multiple intradiscal doses to define the concentration–time relationship, therapeutic window, and optimal administration frequency. The long‐term efficacy and potential side effects of LA or similar lysosome‐modulating agents require comprehensive evaluation.

In conclusion, our findings reveal a close association between lysosomal dysregulation, defective autophagic degradation, cellular senescence, and IVDD progression. The lysosome‐related molecular subtypes and hub genes identified in this study may contribute to improved disease stratification and biomarker development. Furthermore, the protective effects of LA in vitro and in vivo suggest that modulation of lysosomal homeostasis may have therapeutic potential for IVDD. However, additional mechanistic, pharmacological, and translational studies are required before LA can be considered a clinically applicable treatment.

## Author Contributions

Conceptualization: Shixing Chen, Chenglong Ji, and Kaiqiang Sun; Methodology: Yang Yang, Hong Li, and Yixuan Ou; Software: Yang Yang and Yaping Yu; Validation: Yang Yang, Hong Li, and Yixuan Ou; Formal analysis: Yang Yang and Yaping Yu; Investigation: Yang Yang, Hong Li, and Yixuan Ou; Resources: Shixing Chen, Chenglong Ji, and Kaiqiang Sun; Writing – original draft preparation: Yang Yang, Hong Li, and Yixuan Ou; Writing – review and editing: Shixing Chen, Chenglong Ji, and Kaiqiang Sun. All authors read and approved the final manuscript.

## Funding

The authors have nothing to report.

## Ethics Statement

All animal experiments were conducted in strict accordance with the guidelines of our Institutional Animal Care and Use Committee (IACUC) and were approved by the Institutional Review Board of our hospital (2025H012‐A03). The use of human nucleus pulposus tissues was approved by the Ethics Committee of our Hospital (2026Q056‐1). All patients provided written informed consent prior to participation.

## Consent

All the authors in this manuscript have given their consent for publication.

## Conflicts of Interest

The authors declare no conflicts of interest.

## Supporting information


**Table S1:** Lysosome function‐related gene set.

**Table S2:** Raw and multiple‐comparison‐adjusted P values for 28 immune‐cell signatures stratified by lysosome‐related genes.
**Table S3:** Raw and multiple‐comparison‐adjusted P values for 28 immune‐cell signatures control and IVDD group.

**Figure S1:** The representative cell marker for clustering based on single‐cell sequencing.
**Figure S2:** Random permutation analysis of the partial correlation between the lysosomal gene‐signature score and the senescence score. The histogram shows the empirical null distribution of partial correlation coefficients generated from 2000 random permutations while controlling for nCount_RNA. The vertical line indicates the partial correlation coefficient observed in the original dataset. The observed coefficient was significantly greater than that expected under the null distribution. The observed partial correlation coefficient was 0.422, and none of the 2000 permuted coefficients reached or exceeded the observed value, yielding an empirical permutation P value of 0.0005.
**Figure S3:** Volcano plot of differentially expressed genes based on the bulk RNA sequencing.
**Figure S4:** The correlation of hub genes with autophagy‐ and senescence‐related genes.
**Figure S5:** PLD3 modulates lysosomal function and degeneration‐associated phenotypes in H_2_O_2_‐treated nucleus pulposus cells.(A) Representative LysoTracker images of control NPCs and H_2_O_2_‐treated NPCs transfected with control vector, si‐PLD3, or OE‐PLD3. Acidic lysosomal compartments are shown in red and nuclei in blue. Scale bar = 20 μm. (B) Quantification of relative LysoTracker fluorescence intensity. (C) Relative cathepsin B activity normalized to the control group. (D) Relative mRNA expression of *Acan*, *Mmp13*, and *Cdkn1a* determined by RT–qPCR. Data are presented as mean ± SD from three independent experiments (*n* = 3). Statistical significance was assessed using one‐way ANOVA followed by Tukey's multiple‐comparison test. ns, not significant; *p < 0.05, **p < 0.01, ***p < 0.001, and ****p < 0.0001. Abbreviations: NPCs, nucleus pulposus cells; si‐PLD3, PLD3 knockdown; OE‐PLD3, PLD3 overexpression.
**Figure S6:** Cell viability of rat nucleus pulposus treated by LA with different concentrations. *p < 0.05, **p < 0.01, ***p < 0.001 and ****p < 0.000.
**Figure S7:** Lithocholic acid partially restores acidic lysosomal compartments and cathepsin B activity in H_2_O_2_‐treated nucleus pulposus cells. A: Representative fluorescence images of LysoTracker staining in control NPCs and H_2_O_2_‐treated NPCs administered with PBS or lithocholic acid. LysoTracker‐positive acidic lysosomal compartments are shown in red, and nuclei were counterstained with DAPI (blue). Scale bar = 20 μm. B: Quantification of the relative LysoTracker fluorescence intensity. C: Relative cathepsin B activity in the indicated groups, normalized to the control group. Data are presented as mean ± SD from three independent experiments (*n* = 3). Statistical significance was determined using one‐way ANOVA followed by Tukey's multiple‐comparison test. *p < 0.05, ***p < 0.001, and ****p < 0.0001.
**Figure S8:** RT‐qPCR of IVD tissue of rats in sham, IVDD, and LA treatment groups, respectively. *N* = 3. *p < 0.05, **p < 0.01, ***p < 0.001 and ****p < 0.000.

## Data Availability

All data generated or analyzed during this study are included in its supplementary information file.
