# Supplementary material for: Lysosomal Dysfunction Is Associated With Intervertebral Disc Degeneration: Multiomics and Machine Learning Identify Molecular Subtypes and Hub Genes
Source: J Gene Med. 2026 Sep 3;28(9):e70109. doi: 10.1002/jgm.70109 (PMC13539544; doi:10.1002/jgm.70109)
Supplement: Supplementary file 1 [file JGM-28-e70109-s002.docx]

**Table S2 Raw and multiple-comparison-adjusted P values for 28 immune-cell signatures stratified by lysosome-related genes.**

| **ssGSEA_set** | **raw_p** | **q_fdr** | **p_bonferroni** |
| --- | --- | --- | --- |
| Activated B cell | 4.98E-07 | 1.07E-06 | 1.39E-05 |
| Activated CD4 T cell | 8.02E-09 | 2.04E-08 | 2.25E-07 |
| Activated CD8 T cell | 6.64E-10 | 2.07E-09 | 1.86E-08 |
| Activated dendritic cell | 1.28E-11 | 5.95E-11 | 3.57E-10 |
| CD56bright natural killer cell | 0.005460763 | 0.006950062 | 0.152901368 |
| CD56dim natural killer cell | 7.93E-05 | 0.000130602 | 0.002220237 |
| Central memory CD4 T cell | 6.38E-08 | 1.49E-07 | 1.79E-06 |
| Central memory CD8 T cell | 4.33E-06 | 8.08E-06 | 0.00012126 |
| Effector memeory CD4 T cell | 0.19123576 | 0.198318566 | 1 |
| Effector memeory CD8 T cell | 3.33E-10 | 1.17E-09 | 9.33E-09 |
| Eosinophil | 0.186045524 | 0.198318566 | 1 |
| Gamma delta T cell | 1.70E-14 | 1.58E-13 | 4.75E-13 |
| Immature B cell | 1.23E-11 | 5.95E-11 | 3.45E-10 |
| Immature dendritic cell | 0.000779573 | 0.001091402 | 0.021828033 |
| Macrophage | 1.58E-15 | 4.43E-14 | 4.43E-14 |
| Mast cell | 2.44E-06 | 4.88E-06 | 6.83E-05 |
| MDSC | 6.09E-13 | 4.26E-12 | 1.71E-11 |
| Memory B cell | 0.207444521 | 0.207444521 | 1 |
| Monocyte | 0.002055486 | 0.002740648 | 0.057553615 |
| Natural killer cell | 7.06E-06 | 1.24E-05 | 0.00019762 |
| Natural killer T cell | 0.000248767 | 0.00038697 | 0.006965466 |
| Neutrophil | 0.160112059 | 0.179325506 | 1 |
| Plasmacytoid dendritic cell | 0.122680263 | 0.143126973 | 1 |
| Regulatory T cell | 6.43E-15 | 9.00E-14 | 1.80E-13 |
| T follicular helper cell | 7.32E-09 | 2.04E-08 | 2.05E-07 |
| Type 1 T helper cell | 4.07E-11 | 1.63E-10 | 1.14E-09 |
| Type 17 T helper cell | 0.000544787 | 0.000802844 | 0.015254036 |
| Type 2 T helper cell | 0.100717879 | 0.12261307 | 1 |

ssGSEA-derived immune-cell signatures are shown with corresponding raw *P* values, false discovery rate (FDR)-adjusted *q* values, and Bonferroni-adjusted *P* values.

**Table S3 Raw and multiple-comparison-adjusted P values for 28 immune-cell signatures control and IVDD group.**

| ssGSEA_set | raw_p | q_fdr | p_bonferroni |
| --- | --- | --- | --- |
| Activated B cell | 0.20301678 | 0.400955565 | 1 |
| Activated CD4 T cell | 0.834816434 | 0.936082426 | 1 |
| Activated CD8 T cell | 0.552490231 | 0.859429249 | 1 |
| Activated dendritic cell | 0.001333762 | 0.009336331 | 0.037345325 |
| CD56bright natural killer cell | 0.847362972 | 0.936082426 | 1 |
| CD56dim natural killer cell | 0.125004538 | 0.291677256 | 1 |
| Central memory CD4 T cell | 0.000387776 | 0.003619242 | 0.010857727 |
| Central memory CD8 T cell | 0.086404006 | 0.277668403 | 1 |
| Effector memeory CD4 T cell | 0.641637536 | 0.930738787 | 1 |
| Effector memeory CD8 T cell | 0.099167287 | 0.277668403 | 1 |
| Eosinophil | 0.295649928 | 0.517387375 | 1 |
| Gamma delta T cell | 0.095845805 | 0.277668403 | 1 |
| Immature B cell | 0.012440261 | 0.049761042 | 0.348327297 |
| Immature dendritic cell | 0.326554643 | 0.537854706 | 1 |
| Macrophage | 0.214797624 | 0.400955565 | 1 |
| Mast cell | 0.748310606 | 0.936082426 | 1 |
| MDSC | 0.000113496 | 0.001588944 | 0.003177887 |
| Memory B cell | 0.797435979 | 0.936082426 | 1 |
| Monocyte | 0.113362918 | 0.288560155 | 1 |
| Natural killer cell | 0.923334417 | 0.936082426 | 1 |
| Natural killer T cell | 0.834816434 | 0.936082426 | 1 |
| Neutrophil | 0.002285285 | 0.012797595 | 0.063987977 |
| Plasmacytoid dendritic cell | 0.664813419 | 0.930738787 | 1 |
| Regulatory T cell | 0.003406842 | 0.015898595 | 0.095391567 |
| T follicular helper cell | 0.885219049 | 0.936082426 | 1 |
| Type 1 T helper cell | 1.46E-05 | 0.000409834 | 0.000409834 |
| Type 17 T helper cell | 0.175603589 | 0.378223115 | 1 |
| Type 2 T helper cell | 0.936082426 | 0.936082426 | 1 |

ssGSEA-derived immune-cell signatures are shown with corresponding raw P values, false discovery rate (FDR)-adjusted q values, and Bonferroni-adjusted P values.
