# Supplementary material for: Lysosomal Dysfunction Is Associated With Intervertebral Disc Degeneration: Multiomics and Machine Learning Identify Molecular Subtypes and Hub Genes
Source: J Gene Med. 2026 Sep 3;28(9):e70109. doi: 10.1002/jgm.70109 (PMC13539544; doi:10.1002/jgm.70109)
Supplement: Supplementary file 2 [file JGM-28-e70109-s003.docx]

Figure S1 The representative cell marker for clustering based on single-cell sequencing.

**Figure S2 Random permutation analysis of the partial correlation between the lysosomal gene-signature score and the senescence score.** The histogram shows the empirical null distribution of partial correlation coefficients generated from 2,000 random permutations while controlling for nCount_RNA. The vertical line indicates the partial correlation coefficient observed in the original dataset. The observed coefficient was significantly greater than that expected under the null distribution. The observed partial correlation coefficient was 0.422, and none of the 2,000 permuted coefficients reached or exceeded the observed value, yielding an empirical permutation P value of 0.0005.

**Figu****re S3 Volcano plot of differentially expressed genes based on the bulk RNA sequencing.**

**Figure S4 The correlation of hub genes with autophagy- and senescence-related genes.**

**Figure S5** **PLD3 modulates lysosomal function and degeneration-associated phenotypes in H₂O₂-treated nucleus pulposus cells.**
(A) Representative LysoTracker images of control NPCs and H₂O₂-treated NPCs transfected with control vector, si-PLD3, or OE-PLD3. Acidic lysosomal compartments are shown in red and nuclei in blue. Scale bar = 20 μm. (B) Quantification of relative LysoTracker fluorescence intensity. (C) Relative cathepsin B activity normalized to the control group. (D) Relative mRNA expression of *Acan*, *Mmp13*, and *Cdkn1a* determined by RT–qPCR. Data are presented as mean ± SD from three independent experiments (n = 3). Statistical significance was assessed using one-way ANOVA followed by Tukey’s multiple-comparison test. ns, not significant; *p < 0.05, **p < 0.01, ***p < 0.001, and ****p < 0.0001. Abbreviations: NPCs, nucleus pulposus cells; si-PLD3, PLD3 knockdown; OE-PLD3, PLD3 overexpression.

**Figure S6 Cell viability of rat nucleus pulposus treated by LA with different concentrations.** *p < 0.05, **p < 0.01, ***p < 0.001 and ****p < 0.000.

**Figure S7 Lithocholic acid partially restores acidic lysosomal compartments and cathepsin B activity in H₂O₂-treated nucleus pulposus cells.** A: Representative fluorescence images of LysoTracker staining in control NPCs and H₂O₂-treated NPCs administered with PBS or lithocholic acid. LysoTracker-positive acidic lysosomal compartments are shown in red, and nuclei were counterstained with DAPI (blue). Scale bar = 20 μm. B: Quantification of the relative LysoTracker fluorescence intensity. C: Relative cathepsin B activity in the indicated groups, normalized to the control group. Data are presented as mean ± SD from three independent experiments (n = 3). Statistical significance was determined using one-way ANOVA followed by Tukey’s multiple-comparison test. *p < 0.05, ***p < 0.001, and ****p < 0.0001.

**Figure S8 RT-qPCR of IVD tissue of rats in sham, IVDD, and LA treatment groups, respectively.** N=3. *p < 0.05, **p < 0.01, ***p < 0.001 and ****p < 0.000.
